# Glymphatic clearance as revealed by diffusion tensor imaging along the perivascular space (DTI‐ALPS) is associated with Alzheimer's disease neuropathology and periodic rsEEG alpha rhythms in mild cognitive impairment participants

**DOI:** 10.1002/dad2.70384

**Published:** 2026-06-11

**Authors:** Susanna Lopez, Claudio Del Percio, Roberta Lizio, Giuseppe Noce, Matteo Carpi, Dario Arnaldi, Francesco Famà, Matteo Pardini, Federico Massa, Franco Marinozzi, Fabiano Bini, Giorgia Treves, Andrea Soricelli, Marco Salvatore, Franco Giubilei, Laura Ziccardi, Bahar Güntekin, Görsev Yener, Raffaele Ferri, Bartolo Lanuzza, Fabrizio Stocchi, Laura Vacca, Chiara Coletti, Francesco Infarinato, Paola Romano, Moira Marizzoni, Giovanni B. Frisoni, Paolo Barone, Arianna Cappiello, Sofia Cuoco, Laura Bonanni, Anita D'Anselmo, Angelo Antonini, Eleonora Fiorenzato, Simone Cauzzo, Roberta Biundo, Fabrizia D'Antonio, Maria Francesca De Pandis, Simone Marziali, Giuseppe Bruno, Filippo Carducci, Claudio Babiloni

**Affiliations:** ^1^ Department of Physiology and Pharmacology “Vittorio Erspamer,” Sapienza University of Rome Rome Italy; ^2^ Oasi Research Institute ‐ IRCCS Troina Italy; ^3^ IRCCS Synlab SDN Naples Italy; ^4^ Dipartimento di Neuroscienze Oftalmologia, Genetica, Riabilitazione e Scienze Materno‐infantili (DiNOGMI) Università di Genova Genova Italy; ^5^ IRCCS Ospedale Policlinico San Martino Genova Italy; ^6^ Department of Mechanical and Aerospace Engineering Sapienza University of Rome Rome Italy; ^7^ Department of Medical Movement and Well‐being Sciences University of Naples Parthenope Naples Italy; ^8^ Department of Neuroscience Mental Health, and Sensory Organs Sapienza University of Rome Rome Italy; ^9^ Department of Biophysics School of Medicine Istanbul Medipol University İstanbul Turkey; ^10^ Research Institute for Health Sciences and Technologies (SABITA) Neuroscience Research Center Istanbul Medipol University İstanbul Turkey; ^11^ Department of Neurology Faculty of Medicine Dokuz Eylül University İzmir Turkey; ^12^ IBG: International Biomedicine and Genome Center İzmir Turkey; ^13^ IRCCS San Raffaele Roma Rome Italy; ^14^ San Raffaele Open University Rome Italy; ^15^ Biological Psychiatry Unit IRCCS Istituto Centro San Giovanni di Dio Fatebenefratelli Brescia Italy; ^16^ Memory Center Department of Rehabilitation and Geriatrics University Hospitals and University of Geneva Geneva Switzerland; ^17^ Laboratory of Neuroimaging of Aging (LANVIE) University of Geneva Geneva Switzerland; ^18^ Department of Medicine Surgery and Dentistry “Scuola Medica Salernitana,” Neuroscience Section University of Salerno Baronissi Italy; ^19^ Department of Aging Medicine and Sciences University “G. d'Annunzio” of Chieti‐Pescara Chieti Italy; ^20^ IRCCS San Camillo Hospital Venice Italy; ^21^ Parkinson and Movement Disorders Unit, Study Center for Neurodegeneration (CESNE) Department of Neuroscience University of Padua Padua Italy; ^22^ Department of General Psychology University of Padua Padua Italy; ^23^ Department of Human Neurosciences Sapienza University of Rome Rome Italy; ^24^ IRCCS San Raffaele Roma, Cassino site Cassino Italy; ^25^ Department of Human Science and Promotion of Quality of Life San Raffaele Rome University Rome Italy

**Keywords:** Alzheimer's disease, diffusion tensor imaging along the perivascular space, mild cognitive impairment, periodic and aperiodic components of resting state encephalogram power spectral density, resting‐state electroencephalographic rhythms, spectral parametrization

## Abstract

**INTRODUCTION:**

We evaluated whether the brain glymphatic drainage function estimated by the diffusion tensor imaging along the perivascular space (DTI‐ALPS) index relates to white matter (WM) integrity, Alzheimer's disease (AD) neuropathology, resting‐state electroencephalogram (rsEEG) alpha rhythms underpinning quiet vigilance, and cognitive decline in mild cognitive impairment (MCI).

**METHODS:**

Clinical, neuroimaging, and rsEEG data were analyzed in matched mild cognitive impairment due to AD (ADMCI) and MCI not due to AD (noADMCI) participants. DTI‐ALPS index and aperiodic and periodic components of the rsEEG power spectra were calculated following standard pipelines.

**RESULTS:**

Lower DTI‐ALPS index was associated with higher AD neuropathology and WM lesions, lower periodic rsEEG alpha rhythms, and worse cognition in patients with ADMCI and noADMCI as a whole population, with the ADMCI (over noADMCI) group showing lower DTI‐ALPS index, greater AD neuropathology, and lower periodic rsEEG alpha rhythms.

**CONCLUSIONS:**

The DTI‐ALPS index may capture glymphatic system impairment linked to AD neuropathology, vigilance dysfunction, and cognitive decline in MCI.

## Highlights

- Prodromal patients with Alzheimer's disease (AD) showed low brain glymphatic drainage.
- Low glymphatic drainage was associated with AD neuropathology, white matter lesions, and worse cognition.
- Low glymphatic drainage may impact the neurophysiological alpha oscillating processes in quiet vigilance.

## 1 BACKGROUND

Impaired clearance of amyloid beta (Aβ) 1–42 and hyperphosphorylated tau is a key pathogenic mechanism for Alzheimer's disease (AD), driving inflammation, neurodegeneration, and cognitive decline from mild cognitive impairment (ADMCI) to dementia (ADD). ^1^, ^2^ Alongside blood–brain barrier transport and enzymatic degradation, the glymphatic system contributes to remove parenchymal Aβ1–42 and tau proteins via cerebrospinal–interstitial fluid (CSF–ISF) exchange mediated by astrocytic aquaporin‐4 channels. ^3^, ^4^, ^5^, ^6^, ^7^, ^8^, ^9^

Diffusion tensor imaging analysis along the perivascular space (DTI‐ALPS^10^, ^11^) overcomes contrast agents’ injection limitations by measuring water diffusivity within the perivascular spaces (PVSs) of the major white matter (WM) tracts at the level of the lateral ventricular bodies. Patients with AD exhibit reduced DTI‐ALPS index, correlated with higher Aβ1–42 and tau burdens, cortical atrophy in AD signature regions, severe white matter lesions (WMLs), poorer cognition, and enlarged PVS.^5^, ^6^, ^7^ ^;^ ^12^, ^13^, ^14^, ^15^, ^16^ Low DTI‐ALPS indexes precede and predict overt Aβ1–42 accumulation, neurodegeneration, and clinical progression.^5^, ^17^

The glymphatic system is primarily active during sleep when the CSF–ISF exchange increases,^3^, ^18^, ^19^ associated with increased electroencephalography (EEG) delta power and with lower EEG beta power and heart rate, as revealed by electrical impedance spectroscopy.^20^ Yun et al. found that the DTI‐ALPS index increased within 30 minutes after sleep induction in healthy participants.^21^ In patients with AD, low DTI‐ALPS indexes correlate with sleep disturbances and with a faster cognitive decline due to greater Aβ1–42 accumulation,^22^ mediating the impact of sleep disorders on cognition.^23^ Despite the lack of direct biological validation, DTI‐ALPS association with sleep, neuropathology, and neurodegeneration supports its potential role as an indirect marker of glymphatic function.^[^ ^12^ ^]^

Given the relationship between glymphatic function and sleep, we hypothesized that this function may be associated with abnormal cortical arousal and vigilance regulation in patients with ADMCI,^24^ reflected in reduced posterior resting‐state EEG (rsEEG) alpha rhythms (8–12 Hz; ^25^, ^26^). In these patients, rsEEG also reveals diffuse increased delta and theta (< 7 Hz) rhythms, reflecting thalamocortical dysrhythmia^.^ ^27^ Beyond synchronized frequency‐specific cortical neural activity, the aperiodic rsEEG components provide complementary information, modulating the 1/f scaling behavior of the rsEEG spectrum, likely reflecting the underlying excitation–inhibition (E/I) balance and the non‐equilibrium metastable dynamics of cortical networks.^28^

Here, we evaluated whether the DTI‐ALPS index may be associated with WMLs, AD neuropathology, aperiodic and periodic alpha rsEEG components, and cognitive performance in patients with ADMCI compared to those with MCI not due to AD (noADMCI). We hypothesized that glymphatic dysfunction may be associated with aperiodic components that reflect the effects of AD neuropathology on global E/I balance during quiet wakefulness. It may also be associated with the modulation of neural synchronization in thalamocortical circuits and posterior cortex, as measured by periodic alpha oscillations.

**RESEARCH IN CONTEXT**

1. **Systematic Review**: Previous studies showed that patients with Alzheimer's disease (AD) exhibit reduced glymphatic clearance, quantified by diffusion tensor imaging along the perivascular space (DTI‐ALPS) index, correlated with higher amyloid beta 1–42 and tau burdens, cortical atrophy, white matter lesions (WMLs) and poorer cognitive performance. No studies have explored whether this function, strictly modulated by wake–sleep cycle, may be associated with abnormal cortical arousal and vigilance regulation in patients with prodromal AD, reflected in their reduced posterior resting‐state electroencephalogram (rsEEG) alpha (8–12 Hz) rhythms.
2. **Interpretation**: We demonstrate that DTI‐ALPS and WMLs, possibly reflecting impaired brain glymphatic drainage and vascular integrity, may impact the neurophysiological mechanisms oscillating at dominant alpha frequencies that regulate quiet vigilance in patients with prodromal AD.
3. **Future Directions**: Future studies should cross‐validate and extend these results to determine the DTI‐ALPS index prognostic value in prodromal AD and its impact on the brain neurophysiological oscillatory networks regulating quiet vigilance and the wake–sleep cycle.

## 2 METHODS

### 2.1 Participants

This retrospective study included 53 ADMCI, 29 noADMCI, and 43 cognitively unimpaired older controls (Nold), matched for age, sex, and education from the international PharmaCog and PDWAVES Consortium databases (www.pdwaves.eu; Table S1 in supporting information). The study received approval from the local institutional ethics committee and followed the Code of Ethics of the World Medical Association (Declaration of Helsinki). In Supplementary Methods in supporting information, we report the details of the diagnostic criteria, CSF biomarkers, structural magnetic resonance imaging (MRI; T1‐weighted and fluid‐attenuated inversion recovery), DTI and rsEEG data acquisition and processing, and statistical analyses.

### 2.2 DTI‐ALPS calculation

DTI data from a harmonized acquisition protocol^29^ were processed using MRtrix3 (v3.0.3) and FSL (v6.0.7). Raw diffusion images were denoised using MP‐PCA, corrected for Gibbs ringing, and adjusted for eddy currents and motion.

Diffusion tensors were then fitted to derive fractional anisotropy and directional diffusivity maps. T1‐weighted images were skull stripped and registered to Montreal Neurological Institute space, and the resulting transformations were applied to diffusion maps for spatial normalization. All steps were visually inspected for quality control.

The DTI‐ALPS index provides an indirect measure of water diffusivity along PVSs within major WM tracts orthogonal to the lateral ventricles, particularly the superior corona radiata (SCR) and superior longitudinal fasciculus (SLF). In these regions, diffusivity along the left–right (*x*) axis is considered a surrogate marker of perivascular fluid transport, with higher DTI‐ALPS values interpreted as reflecting greater putative glymphatic activity. Although it does not directly measure glymphatic flow, DTI‐ALPS has been proposed as an indirect imaging marker of perivascular diffusivity in neurodegenerative disorders.

Spherical regions of interest (ROIs; 5 mm diameter) were placed bilaterally in both hemispheres in the SCR and SLF, using previously published coordinates (Figure S1 in supporting information). Mean diffusivity values along the *x*‐, *y*‐, and *z* axes were extracted from these ROIs for DTI‐ALPS index calculation.^11^

### 2.3 rsEEG recordings, preprocessing, and power spectral density calculation

RsEEG was recorded using standard clinical EEG systems in the morning, with participants instructed to remain awake and relaxed in eyes‐closed conditions, with a 0.03 to 70 Hz acquisition bandpass. Spectral analyses were performed on uniformly preprocessed data using a common 19‐channel 10 to 20 montage, filtered in the 0.1 to 45 Hz range and resampled, when necessary, to a common sampling frequency of 256 Hz. Data were segmented into 2‐second epochs, visually inspected to remove artifacts, and further cleaned using independent component analysis (ICA^30^).

Power spectral density (PSD) was estimated using the EEGLAB function pop_spectopo with a 1‐second Hanning window without overlap, yielding a frequency sampling of ≈ 1 Hz. Spectral analyses were restricted to the 1 to 40 Hz range. The resulting absolute PSD values were processed using the EEGLAB plugin specparam (https://github.com/bfbarry/EEGLAB‐specparam/), which models the power spectrum as the sum of an aperiodic (1/f‐like) component and periodic oscillatory peaks in log‐log space. The aperiodic component was characterized by its exponent (χ), reflecting the slope of the spectrum, and offset, corresponding to the intercept of the aperiodic component in the log‐log space.

Oscillatory peaks were identified as deviations from the aperiodic component and modeled using Gaussian functions, providing estimates of center frequency, power, and bandwidth. As peak parameters were derived from continuous parametric fits, they were not constrained by the discrete frequency sampling of the PSD. This approach allows sub‐bin frequency precision despite discrete spectral sampling. The individual alpha frequency peak (IAFp^31^) was defined as the center frequency of the most prominent oscillatory peak as identified by the spectral parameterization procedure within 5 to 15 Hz at posterior electrodes.

The delta (1–3 Hz) and theta (4–7 Hz) frequency bands were defined based on standard fixed frequency ranges.^30^ We further identified the low (from IAFp‐2 Hz to IAFp; 5 Hz as the lower limit) and the high (from IAFp to IAFp + 2 Hz; 13 Hz as the upper limit) alpha bands. These limits avoid the inclusion of spurious frequencies typically not within the alpha range.

### 2.4 Main statistical analysis

Between‐group differences were assessed using *t* tests/analysis of variance (ANOVA) for normally ‐distributed continuous variables, Mann–Whitney/Kruskal–Wallis tests for Mini‐Mental State Examination (MMSE) score as non‐interval variable, and χ^2^ tests for categorical variables. Associations among DTI‐ALPS, rsEEG, CSF, WML, and neuropsychological measures related to episodic memory/executive function (Logical Memory immediate recall, Logical Memory Test delayed recall, Rey Auditory Verbal Learning Test [RAVLT] immediate recall, RAVLT delayed recall) were assessed using general linear models (linear fit by ordinary least squares [OLS]) including the group and group × predictor interactions. Significant interactions were further explored using simple effects analyses with false discovery rate (FDR) correction. Variables violating normality assumptions were log transformed. Outliers were evaluated using an iterative Grubbs test (*p* < 0.001). Statistical analyses were conducted using Jamovi (v2.5.6) and MATLAB (R2024b).

## 3 RESULTS

### 3.1 Genetic, CSF Aβ1–42/tau, structural MRI, and neuropsychological variables in noADMCI and ADMCI groups

Table 1 summarizes the demographic, clinical, genetic, CSF Aβ1–42 and tau, MRI markers, and neuropsychological scores, together with the results of their statistical comparisons in the ADMCI and noADMCI participants. Significant differences were found for the apolipoprotein E genotyping (*p* = 0.00001), the AD neuropathology (*p* = 0.0001), the Logical Memory test immediate recall (*p* = 0.001), with more abnormal values in the ADMCI group compared to the noADMCI group.

**TABLE 1:** Mean values (± standard error of the mean) of the demographic (age, sex, and education), clinical (MMSE, GDS, CDR, HIS), genetic (*APOE*), CSF Aβ1‐42 and tau, MRI markers, and neuropsychological scores markers, together with the results of their statistical comparisons in the ADMCI and noADMCI participants.

Demographic, genetic (*APOE*), cerebrospinal fluid (CSF) Aβ1‐42 and tau, MRI markers, and neuropsychological scores in ADMCI and noADMCI groups (mean ± SE)			
	ADMCI	noADMCI	Statistical analysis
**Demographic markers**			
*N*	53	29	–
*Age (years)*	70.5 ± 0.9	69.0 ± 1.4	*t* test: *p* = 0.267
*Sex (F/M, %)*	29/24, 45%	20/9, 31%	χ^2^ test: *p* = 0.208
*Education (years)*	11.3 ± 0.6	10.1 ± 0.8	*t* test: *p* = 0.233
**Clinical markers**			
Mini‐Mental State Examination	25.1 ± 0.2	25.5 ± 0.4	*t* test: *p* = 0.531
Geriatric Depression Scale	2.9 ± 0.2	2.8 ± 0.4	*t* test: *p* = 0.760
Clinical Dementia Rating	0.5 ± 0.0	0.5 ± 0.0	*t* test: *p* = NaN
Hachinski Ischemic Score	0.8 ± 0.1	1.0 ± 0.1	*t* test: *p* = 0.278
**Genetic markers**			
*APOE* ε4 (%)	79.0%	7.0%	**χ^2^ test: *p* = 00001**
**CSF Aβ1‐42 and tau markers (Log10 transformed)**			
CSF Aβ1‐42 (pg/ml)	2.66 ± 0.185	2.96 ± 0.243	***t* test: *p* = 0.0005**
CSF p‐tau (pg/ml)	1.87 ± 0.233	1.68 ± 0.262	***t* test: *p* = 0.0005**
CSF t‐tau (pg/ml)	2.70 ± 0.282	2.43 ± 0.396	***t* test: *p* = 0.001**
CSF Aβ1‐42/p‐tau	0.866 ± 0.0244	1.31 ± 0.0256	***t* test: *p* = 0.0001**
**MRI markers**			
Normalized WM volume	0.290 ± 0.04	0.301 ± 0.05	*t* test: *p* = 0.093
Normalized GM volume	0.386 ± 0.05	0.381 ± 0.05	*t* test: *p* = 0.081
Normalized hippocampal volume	0.00197 ± 0.0001	0.00219 ± 0.0001	*t* test: *p* = 0.123
Normalized amygdala volume	7.71E‐4 ± 0.0001	8.62E‐4 ± 0.0001	*t* test: *p* = 0.080
Precuneus thickness (cm, *Log10 transformed)*	0.715 ± 0.004	0.728 ± 0.004	*t* test: *p* = 0.154
Parietal thickness (cm *Log10 transformed)*	0.965 ± 0.004	0.981 ± 0.004	*t* test: *p* = 0.060
Temporal thickness (cm, *Log10 transformed)*	1.05 ± 0.005	1.06 ± 0.005	*t* test: *p* = 0.472
Mean cortical thickness (cm, *Log10 transformed)*	0.743 ± 0.003	0.748 ± 0.003	*t* test: *p* = 0.677
WM T2‐lesions (mm^3^, Log10 transformed)	3.14 ± 0.7	2.97 ± 0.7	*t* test: *p* = 0.304
WM T1‐hypointensities (mm^3^, Log10 transformed)	3.38 ± 0.3	3.33 ± 0.4	*t* test: *p* = 0.532

Neuropsychological scores				
	Cut‐off of abnormality	Mean ± SE (%subjects with abnormal score)	Mean ± SE (%subjects with abnormal score)	*t test*
Logical Memory Test Immediate recall	*< 13*	7.1 ± 0.8 SE 86.0%	11.4 ± 1.3 SE 57.1%	***p* = 0.001**
Logical Memory Test delayed recall	*< 12*	4.3 ± 0.5 SE 98.0%	5.8 ± 0.6 SE 92.9%	*p* = 0.062
RAVLT immediate recall	*< 28.53*	31.0 ± 1.4 SE 42.0%	33.1 ± 1.4 SE 32.1%	*p* = 0.328
RAVLT delayed recall	*< 4.69*	3.9 ± 0.5 SE 66.0%	4.5 ± 0.6 SE 53.6%	*p* = 0.498
Trail Making Test B‐A	*≥ 187*	140.0 ± 10.1 SE 30.0%	104.1 ± 14.8 SE 9.1%	*p* = 0.052
Letter fluency	*< 17*	32.9 ± 1.7 SE 8.0%	29.6 ± 1.4 SE 17.9%	*p* = 0.262
Letter category	*< 25*	32.6 ± 1.8 SE 39.3%	30.2 ± 1.7 SE 28.0%	*p* = 0.454
Benton Naming Test	*n.a*.	22.8 ± 0.5 SE	22.6 ± 0.9 SE	*p* = 0.831

*Notes*: For the neuropsychological scores, the cut‐off scores for abnormality are reported. The variables undergoing to log10 transformation are specified, as well as normalized or ratio variables (dimensionless).

Abbreviations: Aβ, amyloid beta; AD, Alzheimer's disease; ADMCI, mild cognitive impairment due to Alzheimer's disease; *APOE*, apolipoprotein E; CDR, Clinical Dementia Rating; CSF, cerebrospinal fluid; GDS, Geriatric Depression Scale; GM, gray matter; HIS, Hachinski Ischemic Score; MMSE, Mini‐Mental State Examination; MRI, magnetic resonance imaging; n.a., not applicable; noADMCI, mild cognitive impairment not due to Alzheimer's disease; p‐tau, phosphorylated tau; RAVLT, Rey Auditory Verbal Learning Task; SE, standard error; t‐tau, total tau; WM, white matter.

### 3.2 DTI‐ALPS index in noADMCI and ADMCI participants

A significant two‐way ANOVA interaction (*F*[1, 80] = 5.75; *p* = 0.019; Figure S2 in supporting information) between the group (ADMCI, noADMCI) and ROI (left, right) factors was observed. The ADMCI group was characterized by lower left and right DTI‐ALPS indexes (*p* = 0.038 and *p* = 0.004, respectively, Duncan post hoc FDR corrected) compared to the noADMCI group. In the following statistical analysis, we considered the average between left and right DTI‐ALPS index in relation to the other global variables (WML, AD neuropathology, rsEEG, and cognition) considered in the present study.

### 3.3 Association between WML from T1‐hypointensities and global DTI‐ALPS

**FIGURE 1:**
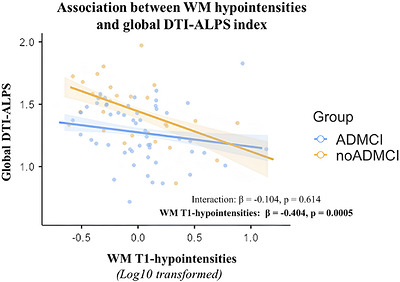
Association between WMLs as revealed by T1‐hypointensities and the global DTI‐ALPS index. The scatterplots illustrate the association between the WML (as revealed from T1‐hypointensities) and group (as predictors) and the global DTI‐ALPS index (target variable; estimated marginal means), as revealed by GLM, in the ADMCI and noADMCI participants. Only the main effect of the WML was statistically significant (standardized β = –0.404, *p* = 0.0005). Standard error of the mean is depicted as shadowed area. AD, Alzheimer's disease; ADMCI, mild cognitive impairment due to Alzheimer's disease; DTI‐ALPS, diffusion tensor imaging along the perivascular space index; GLM, general linear regression model; noADMCI, mild cognitive impairment not due to Alzheimer's disease; WM, white matter; WML, white matter lesion.

Only the main effect of the WML was significant (standardized β = –0.404, *p* = 0.0005; Figure 1). As expected, higher DTI‐ALPS values were associated with fewer WMLs as revealed by T1‐hypoinensities in all the MCI participants.

### 3.4 Association between Aβ1–42/phosphorylated tau neuropathology and global DTI‐ALPS

**FIGURE 2:**
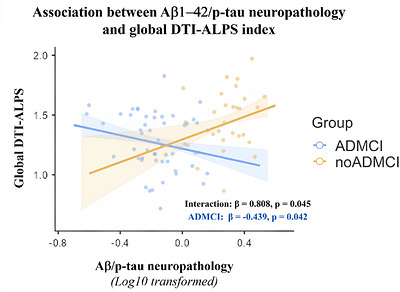
Association between Aβ1−42/p‐tau neuropathology and the global DTI‐ALPS index. The scatterplots illustrate the association between the Aβ1−42/p‐tau neuropathology and group (as predictors) and the global DTI‐ALPS index (target variable; estimated marginal means), as revealed by GLM, in the ADMCI and noADMCI participants. A statistically significant interaction effect between the Aβ1–42/p‐tau neuropathology and the group factors was observed (standardized β = 0.808, *p* = 0.045). No significant FDR‐corrected group difference was observed (*p* = 0.441). Simple effect revealed a slight statistically significant effect only for the ADMCI (standardized *β* = –0.439, *p* = 0.042) group, while no effect was observed for the noADMCI (standardized β = 0.314, *p* = 0.354) group. Standard error of the mean is depicted as shadowed area. Aβ, amyloid beta; Aβ1–42/p‐tau, ratio between the amyloid beta 1–42 and phosphorylated tau markers in the cerebrospinal fluid; AD, Alzheimer's disease; ADMCI, mild cognitive impairment due to Alzheimer's disease; DTI‐ALPS, diffusion tensor imaging along the perivascular space index; FDR, false discovery rate; GLM, general linear regression model; noADMCI, mild cognitive impairment not due to Alzheimer's disease; p‐tau, phosphorylated tau.

A significant interaction between the Aβ1–42/phosphorylated tau (p‐tau) neuropathology and the group factors was observed (standardized β = 0.808, *p* = 0.045; Figure 2). Group simple effect revealed a slight statistically significant effect only for the participants in the ADMCI group (standardized β = –0.439, *p* = 0.042), while no effect was observed for the noADMCI group (standardized *β* = 0.314, *p* = 0.354). These results suggest that clinically relevant AD neuropathology may interfere with glymphatic function, even inverting the physiological clearance of toxic solutes, such as p‐tau, through the CSF–ISF flow.

No significant interaction was observed including the Aβ1–42 (standardized *β* = 0.091, *p* = 0.768) or p‐tau (standardized *β* = –0.422, *p* = 0.102) neuropathology and the group as predictors (Figure S3 in supporting information).

### 3.5 Periodic and aperiodic components of the rsEEG PSD

Both MCI groups showed an evident reduction of the corrected rsEEG PSD within the alpha frequency band compared (8–12 Hz) to the Nold group, with a prominent reduction for ADMCI participants compared to noADMCI participants (Figure S4 in supporting information).

The mean IAFp was 8.8 Hz (6.1–10.3 Hz; ± 0.18 standard error [SE]) in the Nold, 8.4 Hz (6.1–12.6 Hz; ± 0.25 SE) in the ADMCI, and 8.9 Hz (6.1–12.6 Hz; ± 0.3 SE) in the noADMCI group. No significant differences were found among the three groups (ANOVA; *p* = 0.331; Figure S5 in supporting information) nor between the ADMCI and noADMCI groups (*t* test; *p* = 0.116). Overall spectral parameterization performance was excellent (mean *R* ^2^ = 0.99; range: 0.96–0.998; mean fit error = 0.05; range: 0.02–0.10). The ADMCI and noADMCI groups did not differ for the mean *R* ^2^ (*p* = 0.190), mean fit error (*p* = 0.465), the artifact‐free epochs (*p* = 0.242), and number of suppressed noisy independent components (*p* = 0.954).

No statistically significant ANOVA main (group) or interaction (group x ROI) effects were observed for the exponent (*p* = 0.081 and *p* = 0.438, respectively) and offset variables (*p* = 0.201 and *p* = 0.155, respectively; Figure S6 in supporting information).

A statistically significant three‐way ANOVA interaction (*F*[12, 732] = 5.95, *p* = 0.005) among the factors group (Nold, ADMCI, noADMCI), band (delta, theta, low alpha, high alpha), and ROI (frontal, central, parieto‐occipital). Compared to the Nold, the ADMCI and noADMCI groups were characterized by lower periodic parieto‐occipital high alpha rsEEG PSD (*p* < 0.05 FDR corrected Duncan post hoc testing). Furthermore, compared to the Nold, the ADMCI group was characterized by lower periodic widespread low and high alpha rsEEG PSD (*p* < 0.05 FDR‐corrected Duncan post hoc testing; Figure S7 in supporting information).

As 20 participants (14 ADMCI, 6 noADMCI) exhibited an IAFp < 7.5 Hz, we included a control analysis comparing the eyes‐closed to eyes‐open recordings to evaluate the reactivity to eye opening (i.e., desynchronization) of low alpha rsEEG PSD, justifying the definition as low alpha sub‐band (typically, “reactive” to eyes opening) and not theta band (unreactive). Periodic global rsEEG PSD during eyes‐open was lower compared to eyes‐closed condition for both sub‐bands (low alpha: *p* = 0.002, high alpha: *p* = 0.001; FDR‐corrected Duncan post hoc testing; Figure S8 in supporting information).

**FIGURE 3:**
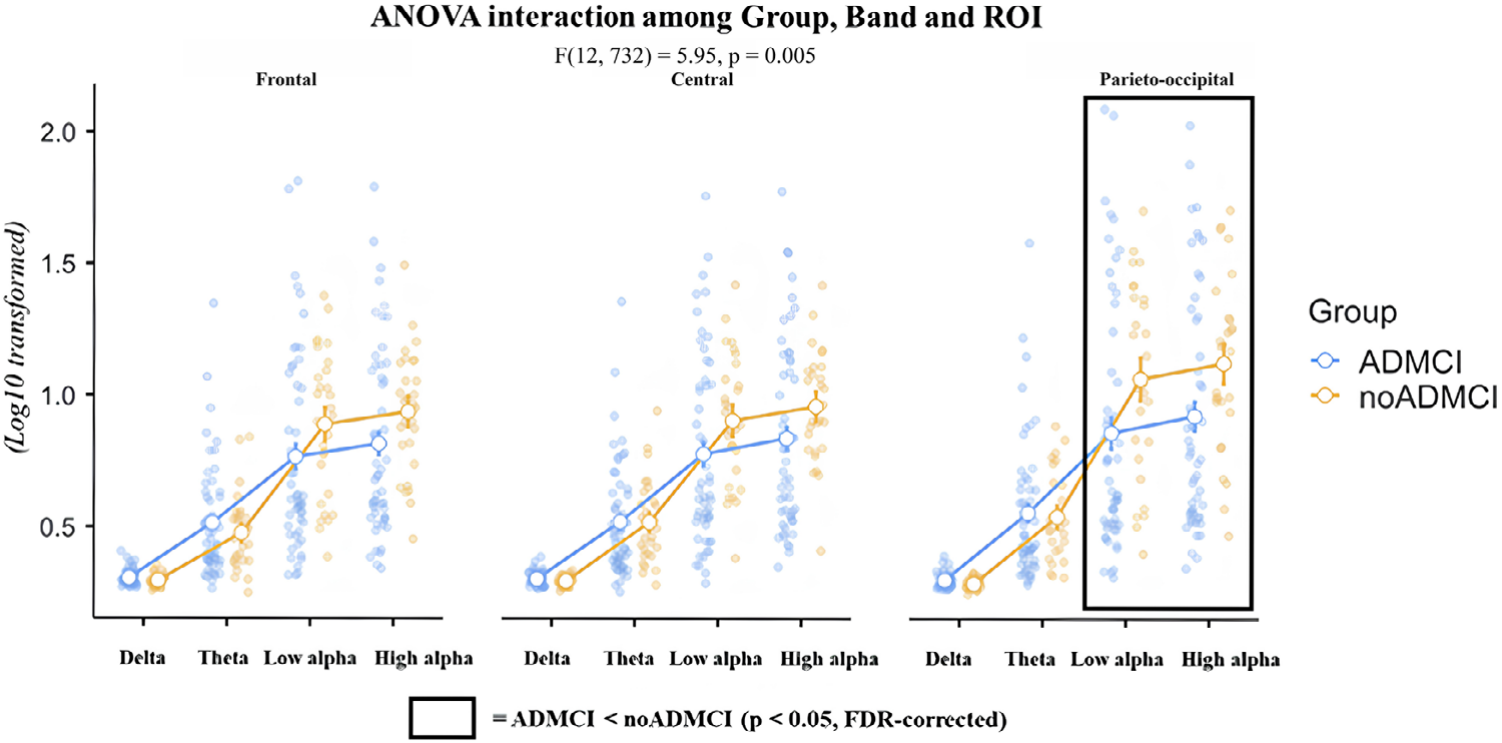
Regional periodic components of the rsEEG PSD. Mean values (± standard error mean, SE) of periodic rsEEG PSD for (1) two groups (ADMCI, noADMCI), (2) three ROIs (frontal, central, parieto‐occipital), and (3) four frequency bands (delta, theta, low alpha, high alpha). The ANOVA showed a statistically significant three‐way interaction (*F*[6, 480] = 3.78, *p* = 0.001) among the factors group (ADMCI, noADMCI), band (delta, theta, low alpha, high alpha), and ROI (frontal, central, parieto‐occipital). Compared to the noADMCI groups, the ADMCI group was characterized by lower periodic parieto‐occipital low and high alpha rsEEG PSD, as revealed by Duncan post hoc testing (*p* < 0.05 FDR corrected). The aperiodic‐corrected rsEEG PSD were used to identify the individual alpha frequency peak (IAFp) defined as the frequency showing the maximum corrected PSD value between 5 and 15 Hz from posterior electrodes (P3, Pz, P4, P7, P8, O1, O2). The frequency bands were: delta (1–3 Hz), theta (4–7 Hz), and low and high alpha. These alpha sub‐bands were identified as follows: the low (from IAFp‐2 Hz to IAFp; 5 Hz as the lower limit) and the high (from IAFp to IAFp + 2 Hz; 13 Hz as the upper limit) alpha bands. The aperiodic‐corrected rsEEG PSD were calculated in the following scalp ROIs: frontal (Fp1, Fp2, F3, Fz, F4, F7, F8), central (T7, T8, C3, Cz, C4), and posterior (P3, Pz, P4, P7, P8, O1, O2). AD, Alzheimer's disease; ADMCI, mild cognitive impairment due to Alzheimer's disease; ANOVA, analysis of variance; FDR, false discovery rate; noADMCI, mild cognitive impairment not due to Alzheimer's disease; rsEEG, resting‐state electroencephalography; PSD, power spectral density; ROI, region of interest.

To address the study hypothesis, Figure 3 illustrates the results of an analysis focused solely on the ADMCI and noADMCI groups. There was a significant three‐way ANOVA interaction (*F*[6, 480] = 3.78, *p* = 0.001) among the factors group (ADMCI, noADMCI), band (delta, theta, low alpha, high alpha), and ROI (frontal, central, parieto‐occipital). Compared to the noADMCI groups, the ADMCI group was characterized by lower periodic parieto‐occipital low and high alpha rsEEG PSD (*p* < 0.05 FDR‐corrected Duncan post hoc testing), reflecting abnormalities in cortical neural synchronization generating periodic posterior alpha rhythms in prodromal AD.

### 3.6 Association between global DTI‐ALPS and periodic components of the rsEEG PSD

**FIGURE 4:**
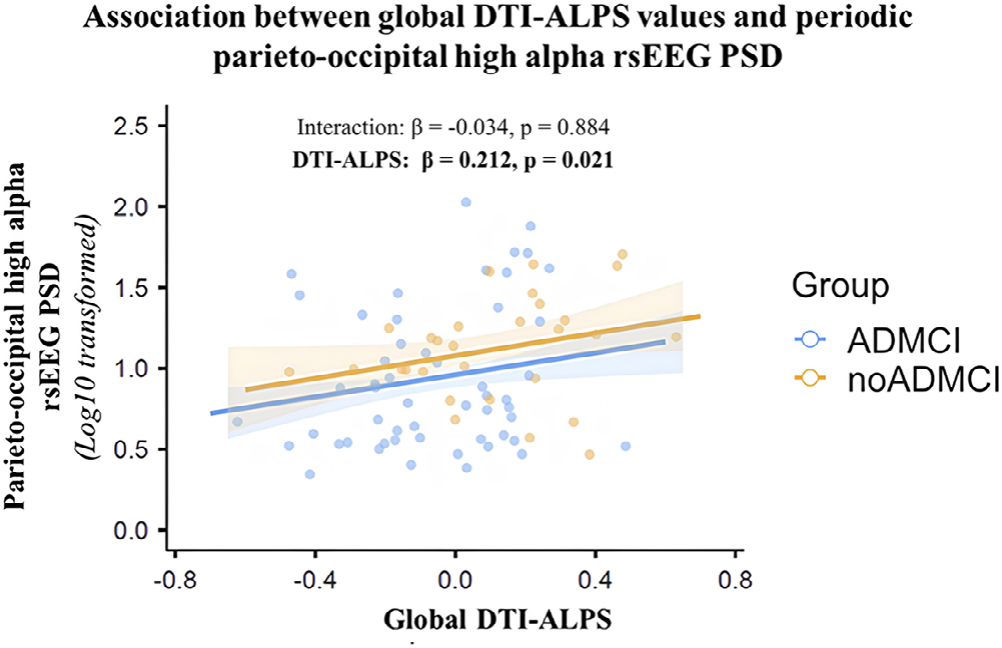
Association between global DTI‐ALPS and periodic rsEEG PSD. The scatterplots illustrate the association between the global DTI‐ALPS index and group (as predictors) and the periodic parieto‐occipital high alpha as revealed by GLM, in the ADMCI and noADMCI participants. Only the main effect of the global DTI‐ALPS index was statistically significant on the parieto‐occipital high alpha rsEEG PSD (standardized β = 0.212, *p* = 0.021). Standard error of the mean is depicted as shadowed area. ADMCI, mild cognitive impairment due to Alzheimer's disease; DTI‐ALPS, diffusion tensor imaging along the perivascular space index; GLM, general linear regression model; noADMCI, mild cognitive impairment not due to Alzheimer's disease; rsEEG, resting‐state electroencephalography; PSD, power spectral density.

Figure 4 illustrates the association between the global DTI‐ALPS and the group as predictors and the periodic components of the rsEEG PSD as target variables. Given the results of the previous ANOVA, we focused on the global and parieto‐occipital low and high alpha rsEEG PSD. Only the main effect of the global DTI‐ALPS index was significant on the parieto‐occipital high alpha rsEEG PSD (standardized β = 0.212, *p* = 0.021). As expected, higher DTI‐ALPS values were associated with higher (less abnormal) periodic posterior alpha rsEEG PSD in all the MCI participants.

No significant main effect was observed including the exponent (standardized *β* = 0.123, *p* = 0.371) or offset (standardized *β* = –0.157, *p* = 0.252) aperiodic rsEEG parameters and the group as predictors (Figure S9 in supporting information).

### 3.7 Association between global DTI‐ALPS and memory/executive function

**FIGURE 5:**
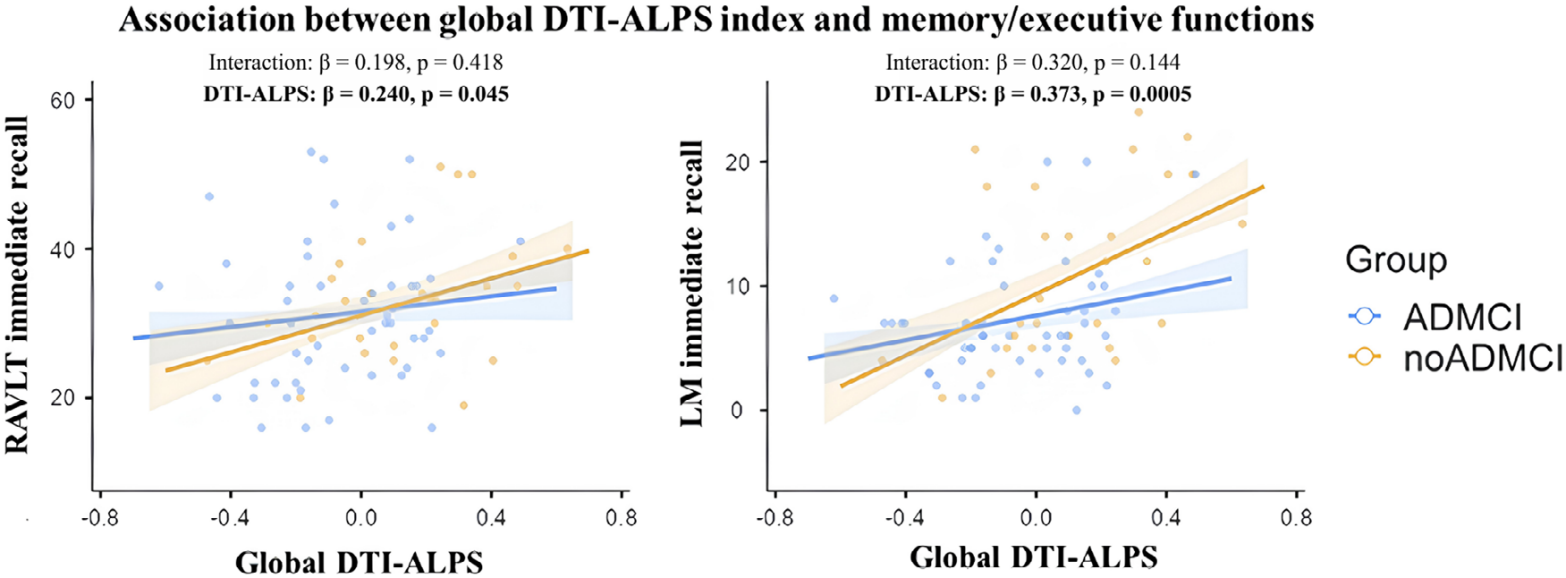
Association between global DTI‐ALPS and memory/executive function. The scatterplots illustrate the association between the global DTI‐ALPS index and group (as predictors) and the memory/executive function (target variable; estimated marginal means), as revealed by GLM, in the ADMCI and noADMCI participants. Only the main effect of the global DTI‐ALPS index was statistically significant on the RAVLT immediate recall (standardized β = 0.240, *p* = 0.045) and on the Logical Memory immediate recall (standardized β = 0.373, *p* = 0.0005). Standard error of the mean is depicted as shadowed area. Legend: ADMCI, mild cognitive impairment due to Alzheimer's disease; DTI‐ALPS, diffusion tensor imaging along the perivascular space index; GLM, general linear regression model; noADMCI, mild cognitive impairment not due to Alzheimer's disease; RAVLT, Rey Auditory Verbal Learning Test.

Only the main effect of the global DTI‐ALPS index was statistically significant on the RAVLT immediate recall (standardized β = 0.240, *p* = 0.045; Figure 5) and on the Logical Memory immediate recall (standardized β = 0.373, *p* = 0.0005). As expected, higher DTI‐ALPS values were associated with higher neuropsychological scores in all the participants with MCI.

### 3.8 Control analysis

For cross‐validation purposes, in the Supplementary Results in supporting information, we performed the spectral parametrization using the absolute PSD values ranging within 1 to 30 Hz and 3 to 30 Hz (Figure S10 in supporting information). We also estimated the DTI‐ALPS index with a different method.

The results confirmed the main findings on periodic alpha rsEEG PSD, of lower DTI‐ALPS index in the ADMCI group (Figure S11 in supporting information) and the associations with fewer WMLs (Figure S12 in supporting information), higher parieto‐occipital periodic high alpha rsEEG PSD (Figure S13 in supporting information), and better cognition (Figure S14 in supporting information), in both groups. Furthermore, we confirmed the different trends in the association between AD neuropathology and global DTI‐ALPS for the ADMCI (negative) and noADMCI (positive) groups (Figure S15 in supporting information).

## 4 DISCUSSION

Here, we evaluated whether the DTI‐ALPS values, proposed to reflect the brain glymphatic drainage system, may be associated with WML, AD neuropathology, aperiodic and periodic rsEEG alpha components, and cognitive performance in patients with ADMCI compared to those with noADMCI. Spectral parametrization disentangled periodic and aperiodic components of rsEEG power spectra, enabling the assessment of global neural dynamics beyond frequency‐specific oscillatory processes, as in recent rsEEG studies of patients with AD.^32^, ^33^, ^34^ We hypothesized that glymphatic dysfunction may be associated with aperiodic components reflecting the effects of AD neuropathology on global E/I balance during quiet wakefulness. It may also be associated with modulation of neural activity synchronization in thalamocortical circuits and the posterior cortex, as measured by periodic alpha oscillations.

### 4.1 DTI‐ALPS is associated with AD neuropathology, WML, periodic alpha rsEEG power, and cognitive performance

Patients with ADMCI exhibited lower DTI‐ALPS values, greater abnormalities in CSF Aβ1–42/p‐tau biomarkers, higher WM abnormalities, and lower posterior aperiodic‐corrected rsEEG alpha power than patients with noADMCI, corroborating previous evidence in the AD continuum.^6^, ^7^, ^8^, ^9^, ^10^, ^11^, ^12^, ^13^, ^14^, ^15^, ^16^, ^17^, ^18^, ^19^ Prodromal AD features may impair glymphatic clearance, potentially driven by perivascular space enlargement or microvascular dysfunction.^15^ Together with white matter abnormalities, this may limit the removal of Aβ1–42 and p‐tau, promoting neuroinflammation and accelerating neurodegeneration.^35^, ^36^

Importantly, our results extend prior literature by showing that, across both MCI groups, higher DTI‐ALPS values were associated with lower WM abnormalities, higher posterior alpha oscillatory activity, and better cognitive performance. This pattern suggests that DTI‐ALPS–related alterations may reflect a neurobiological substrate shared across patients with MCI, linking perivascular dysfunction, large‐scale oscillatory network activity at the typical dominant alpha frequencies underpinning quiet wakefulness, and cognitive decline. This interpretation is consistent with evidence that reduced DTI‐ALPS value is not pathognomonic of AD^10^ and may occur in other neurological conditions,^37^, ^38^ including vascular risk and brain WMLs in cerebral small vessel disease, which are typically present to some extent in older individuals with cognitive decline.^7^, ^39^ These findings support the potential of diagnostic and therapeutic strategies targeting brain glymphatic and vascular systems, extending beyond traditional disease‐specific frameworks.^7^, ^37^, ^39^

No group differences in the rsEEG aperiodic exponent and offset parameters were observed. Although recent studies have proposed that the aperiodic exponent may reflect a global cortical E/I balance and that the offset may index global firing rates and metabolic activity,^40^ these components have been often considered to be neural noise not linked to a specific biological mechanism or higher brain function,^41^ as both may be influenced by residual artifacts, volume conduction, and non‐linear neuron–glia interactions contributing to the population synaptic dynamics and neuron membrane time constants during quiet wakefulness.

The absence of group differences in our sample suggests that these global neural properties may not be primarily affected by glymphatic dysfunction at the prodromal AD stage, or that our sample size may have limited sensitivity to detect subtle effects. Indeed, recent larger rsEEG investigation has reported higher aperiodic components in patients with ADMCI and ADD compared to Nold controls in a cohort of hundreds of participants^32^, ^33^ and compared to patients with frontotemporal dementia.^34^ Grounded in the present findings, an altered brain glymphatic clearance may not primarily and specifically affect cortical neural E/I balance or global neural firing in patients with prodromal AD. However, at later AD stages, meningeal lymphatic dysfunction may affect cortical neural E/I balance in these patients.^42^ The lack of results on the aperiodic components may also be due to a predominant role of tau pathology. As tau pathology reflects greater neurodegeneration, the E/I imbalance concomitant with amyloidosis may be contrasted with tau pathology, which promotes neural silencing. To address this specific issue in the future, the aperiodic rsEEG component should be associated with the topography of the amyloid positron emission tomography markers on larger cohorts.

It can be speculated that the rsEEG spectral periodic components reflecting oscillatory activity in alpha frequencies within posterior cortical networks may better reflect, not necessarily with causality, the impact of an impaired brain glymphatic drainage system on the neurophysiological mechanisms regulating quiet vigilance, especially, but not exclusively, in patients with ADMCI.^32^, ^43^, ^44^ This oscillatory alpha activity depends on the functional integrity of thalamocortical and corticocortical synchronization and long‐range coordination, modulated by subcortical ascending neuromodulatory systems regulating quiet vigilance.^32^, ^45^, ^46^, ^47^, ^48^ One possible explanation is that impaired perivascular fluid transport may contribute to the accumulation of metabolic waste products, neuroinflammatory mediators, and vascular dysfunction, which in turn may alter extracellular ionic homeostasis and large‐scale neuronal synchronization mechanisms supporting alpha oscillations during quiet wakefulness.

However, the present cross‐sectional findings do not allow inference about causal relationships or direct physiological coupling between DTI‐ALPS–related processes and rsEEG dynamics. The impact of inefficient brain glymphatic clearance on these neurophysiological oscillatory mechanisms may also be mediated by related neuroinflammatory processes,^35^, ^36^ driven by metabolic waste products, oxidative stress, and neurovascular uncoupling.^49^ Finally, an alternative explanation is that inefficient brain glymphatic clearance may merely be a concomitant of AD neurodegeneration.

### 4.2 The association between DTI‐ALPS values and CSF Aβ1–42/p‐tau

Higher DTI‐ALPS values were associated with lower AD pathology in patients with noADMCI, as indicated by the higher CSF Aβ1–42/p‐tau ratio, consistent with previous observations in healthy participants and across the AD spectrum.^15^, ^16^, ^17^

A paradoxical negative association was observed between DTI‐ALPS values and the CSF Aβ1–42/p‐tau ratio in patients with ADMCI. This finding apparently challenges the expectation that an enhanced glymphatic function may be beneficial in patients with ADMCI. Indeed, the current results suggest that this paradoxical association in patients with ADMCI is driven by a positive association between DTI‐ALPS values and CSF p‐tau, reflecting a strong chemical gradient that moves p‐tau from the brain parenchyma to the CSF in the lateral ventricles.^50^ In patients with noADMCI, this gradient would be low due to low CSF p‐tau accumulation. Because of its fibrillary nature and tendency to aggregate into plaques, Aβ1–42 may have greater difficulty than p‐tau in moving through extracellular spaces within the glymphatic pathway due to chemical gradients.^50^

Some limitations should be acknowledged (see Supplementary Discussion in supporting information). We use relatively short analysis windows (≈ 1 second), which provide limited spectral resolution, though this is partially mitigated by parametric spectral modeling. Additionally, results may depend on parameterization settings, although standard, widely used configurations were adopted. The DTI‐ALPS index shows limited sensitivity and specificity for glymphatic assessment,^50^ as it primarily reflects bulk Brownian water diffusion rather than dynamic CSF–ISF exchange.^20^ Its signal is strongly confounded by WM microstructural factors (crossing fibers, axonal dispersion, undulations) and the low spatial resolution of DTI. Moreover, the sparse and heterogeneous architecture of perivascular spaces and medullary veins limits the biological interpretability of DTI‐ALPS as a direct marker of glymphatic dysfunction.

## 5 CONCLUSIONS

Patients with ADMCI show reduced DTI‐ALPS values, greater AD neuropathology, and impaired posterior alpha rsEEG activity compared to patients with noADMCI. Across patients with MCI, lower DTI‐ALPS values were associated with greater WM lesions, altered posterior alpha rhythms, and poorer cognition. These findings highlight the relevance of glymphatic and vascular integrity in modulating neurophysiological networks and cognition in prodromal AD. Longitudinal studies are needed to clarify causality and prognostic implications.

## CONFLICT OF INTEREST STATEMENT

None of the authors have potential conflicts of interest or competing financial interests related to the present article. Their contribution to this article reflects only and exclusively their academic expertise. Some of them served or are serving as board or ad hoc reviewers, chief, associate, or handling editors for scientific journals. They declare no editorial interference with the editorial processing of the present article. Furthermore, some of them received honoraria as consultants from industries, pharmaceutical companies, or small‐medium enterprises, but this is irrelevant to the contents of the present article. Author disclosures are available in the Supporting Information.

## CONSENT STATEMENT

The local institutional ethical committees approved the study. All experiments were conducted in accordance with the Code of Ethics of the World Medical Association (Declaration of Helsinki) and the standards established by the local institutional review boards. All participants or their representatives gave written informed consent for the use of their clinical data for research purposes. Participants were recruited without discrimination based on sex, ethnicity, socioeconomic status, or other personal characteristics, ensuring a diverse and representative sample.

## Supporting information




Supporting Information

Supporting Information

## References

[1] Jack CR , Andrews JS , Beach TG , et al. Revised criteria for diagnosis and staging of Alzheimer's disease: Alzheimer's association workgroup. Alzheimer's & Dementia. 2024;20(8):5143‐5169. doi:10.1002/alz.13859 PMC11350039 38934362

[2] Jongsiriyanyong S , Limpawattana P . Mild cognitive impairment in clinical practice: A review Article. Am J Alzheimers Dis Other Demen. 2018;33(8):500‐507. doi:10.1177/1533317518791401 30068225 PMC10852498

[3] Harrison IF , Ismail O , Machhada A , et al. Impaired glymphatic function and clearance of tau in an Alzheimer's disease model. Brain. 2020;143(8):2576‐2593. doi:10.1093/brain/awaa179 32705145 PMC7447521

[4] Jessen NA , Munk ASF , Lundgaard I , Nedergaard M . The glymphatic system: A beginner's guide. Neurochem Res. 2015;40(12):2583‐2599. doi:10.1007/s11064-015-1581-6 25947369 PMC4636982

[5] Huang S , Zhang Y , Guo Y , et al. Glymphatic system dysfunction redicts amyloid deposition, neurodegeneration, and clinical progression in Alzheimer's disease. Alzheimer's & Dementia. 2024;20(5):3251‐3269. doi:10.1002/alz.13789 PMC11095446 38501315

[6] Hong H , Hong L , Luo X , et al, the Alzheimer's Disease Neuroimaging Initiative (ADNI) . The relationship between amyloid pathology, cerebral small vessel disease, glymphatic dysfunction, and cognition: A study based on Alzheimer's disease continuum participants. Res Therapy. 2024;16(1):43. doi:10.1186/s13195-024-01407-w PMC10877805 38378607

[7] Kim M , Song YS , Han K , Bae YJ , Han JW , Kim KW . Impaired glymphatic flow on diffusion tensor MRI as a marker of neurodegeneration in Alzheimer's disease: correlation with gray matter volume loss and cognitive decline independent of cerebral amyloid deposition. Journal of Alzheimer's Disease. 2024;99(1):279‐290. doi:10.3233/JAD-231131 38669532

[8] Li H , Yao Q , Huang X , Yang X , Yu C . The role and mechanism of Aβ clearance dysfunction in the glymphatic system in Alzheimer's disease comorbidity. Front Neurol. 2024;15:1474439. doi:10.3389/fneur.2024.1474439 39655162 PMC11626247

[9] Rasmussen MK , Mestre H , Nedergaard M . The glymphatic pathway in neurological disorders. The Lancet Neurology. 2018;17(11):1016‐1024. doi:10.1016/S1474-4422(18)30318-1 30353860 PMC6261373

[10] Taoka T , Masutani Y , Kawai H , et al. Evaluation of glymphatic system activity with the diffusion MR technique: diffusion tensor image analysis along the perivascular space (DTI‐ALPS) in Alzheimer's disease cases. Jpn J Radiol. 2017;35(4):172‐178. doi:10.1007/s11604-017-0617-z 28197821

[11] Botta D , Hutuca I , Ghoul EE , et al. Emerging non‐invasive MRI techniques for glymphatic system assessment in Neurodegenerative disease. J Neuroradiol. 2025;52(3):101322. doi:10.1016/j.neurad.2025.101322 39894249

[12] Hsu J , Wei Y , Toh CH , et al. magnetic resonance images implicate that glymphatic alterations mediate cognitive dysfunction in Alzheimer disease. Annals of Neurology. 2023;93(1):164‐174. doi:10.1002/ana.26516 36214568 PMC10091747

[13] Chen Q , Ge D , Xu X , et al. Glymphatic function associates with Alzheimer's disease—signature region volumes, plasma biomarkers and white matter hyperintensity progression in cognitively unimpaired older adults. Age and Ageing. 2025;54(6):afaf141. doi:10.1093/ageing/afaf141 40459343 PMC12131235

[14] Schirge PM , Perneczky R , Taoka T , et al. Perivascular space and white matter hyperintensities in Alzheimer's disease: Associations with disease progression and cognitive function. Alz Res Therapy. 2025;17(1):62. doi:10.1186/s13195-025-01707-9 PMC11917016 40098158

[15] Xue H , Bi S , Chen Z , et al. Glymphatic system dysfunction mediates amyloid deposition and cognitive impairment in Alzheimer's disease: a PET/MRI multimodality imaging study. EJNMMI Res. 2025;16(1):2. doi:10.1186/s13550-025-01339-y 41313418 PMC12764736

[16] Zhang X , Wang Y , Jiao B , et al. Glymphatic system impairment in Alzheimer's disease: associations with perivascular space volume and cognitive function. Eur Radiol. 2023;34(2):1314‐1323. doi:10.1007/s00330-023-10122-3 37610441

[17] Jungwon J , Lee JH , Choi C‐H , Lee J . DTI‐ALPS index as a predictor of cognitive decline over 1 year. Neuroradiology. 2025;67(1):163‐170. doi:10.1007/s00234-024-03521-w 39680094

[18] Lim MM , Gerstner JR , Holtzman DM . The sleep–wake cycle and alzheimer's disease: what do we know?. Neurodegener Dis Manag. 2014;4(5):351‐362. doi:10.2217/nmt.14.33 25405649 PMC4257134

[19] Hablitz LM , Plá V , Giannetto M , et al. Circadian control of brain glymphatic and lymphatic fluid flow. Nat Commun. 2020;11(1):4411. doi:10.1038/s41467-020-18115-2 32879313 PMC7468152

[20] Dagum P , Giovangrandi L , Levendovszky SR , et al. A wireless device for continuous measurement of brain parenchymal resistance tracks glymphatic function in humans. Nat Biomed Eng. 2025;9(10):1656‐1676. doi:10.1038/s41551-025-01394-9 40425804 PMC12532611

[21] Yun CS , Sohn CH , Yeon J , et al. Feasibility of observing glymphatic system activity during sleep using diffusion tensor imaging analysis along the perivascular space (DTI‐ALPS) index. Diagnostics (Basel). 2025;15(14):1798. doi:10.3390/diagnostics15141798. Published 2025 Jul 16 40722547 PMC12293424

[22] Khandayataray P , Murthy MK . Exploring the nexus: sleep disorders, circadian dysregulation, and Alzheimer's disease. Neuroscience. 2025;574:21‐41. doi:10.1016/j.neuroscience.2025.03.066 40189132

[23] Shang Y , Yu L , Xing H , et al. Diffusion tensor imaging analysis along the perivascular space (DTI‐ALPS) demonstrates that sleep disorders exacerbate glymphatic circulatory impairment and cognitive impairment in patients with Alzheimer's Disease. NSS. 2024;16:2205‐2215. doi:10.2147/NSS.S496607 PMC11675307 39735385

[24] Hughes SW , Crunelli V . Thalamic mechanisms of EEG alpha rhythms and their pathological implications. Neuroscientist. 2005;11(4):357‐372. doi:10.1177/1073858405277450 16061522

[25] Pfurtscheller G , Lopes da Silva FH . Event‐related EEG/MEG synchronization and desynchronization: basic Principles. Clin Neurophysiol. 1999;110(11):1842‐1857. doi:10.1016/s1388-2457(99)00141-8 10576479

[26] Babiloni C , Blinowska K , Bonanni L , Cichocki A , Haan W , Del Percio C . What electrophysiology tells us about Alzheimer's disease: a window into the synchronization and connectivity of brain neurons. Neurobiol Aging. 2020;85:58‐73. doi:10.1016/j.neurobiolaging.2019.09.008 31739167

[27] Llinás RR , Steriade M . Bursting of Thalamic Neurons and States of Vigilance. Journal of Neurophysiology. 2006;95(6):3297‐3308. doi:10.1152/jn.00166.2006 16554502

[28] Deco G , Kringelbach ML , Jirsa VK , et al. The dynamics of resting fluctuations in the brain: metastability and its dynamical cortical core. Sci Rep. 2017;7(1):3095. doi:10.1038/s41598-017-03073-5 28596608 PMC5465179

[29] Nathan PJ , Lim YY , Abbott R , et al. Association between CSF biomarkers, hippocampal volume and cognitive function in patients with amnestic mild cognitive impairment (MCI). Neurobiology of Aging. 2017;53:1‐10. doi:10.1016/j.neurobiolaging.2017.01.013 28189924

[30] Del Percio C , Lizio R , Lopez Setal . Resting‐state EEG alpha rhythms are related to csf tau biomarkers in prodromal Alzheimer's disease. Int J Mol Sci. 2025;26(1):356. doi:10.3390/ijms26010356 39796211 PMC11720070

[31] Klimesch W . EEG alpha and theta oscillations reflect cognitive and memory performance: a review and analysis. Brain Res Brain Res Rev. 1999;29(2‐3):169‐195. doi:10.1016/s0165-0173(98)00056-3 10209231

[32] Kopčanová M , Tait L , Donoghue Tetal . Resting‐state EEG signatures of Alzheimer's disease are driven by periodic but not aperiodic changes. Neurobiology of Disease. 2024;190:106380. doi:10.1016/j.nbd.2023.106380 38114048

[33] Jaramillo‐Jimenez A , Mantilla‐Ramos Y‐J , Tovar‐Rios DA , et al. Characterizing resting‐state EEG oscillatory and aperiodic activity in Neurodegenerative diseases: a multicentric study. Computers in Biology and Medicine. 2025;197:111080. doi:10.1016/j.compbiomed.2025.111080 40967145

[34] Wang Z , Liu A , Yu J , et al. The effect of aperiodic components in distinguishing Alzheimer's disease from frontotemporal dementia. GeroScience. 2023;46(1):751‐768. doi:10.1007/s11357-023-01041-8 38110590 PMC10828513

[35] Cai Y , Zhang Y , Leng S , et al. The relationship between inflammation, impaired glymphatic system, and neurodegenerative disorders: A vicious cycle. Neurobiology of Disease. 2024;192:106426. doi:10.1016/j.nbd.2024.106426 38331353

[36] Zou K , Deng Q , Zhang H , Huang C . Glymphatic system: A gateway for Neuroinflammation. Neural Regeneration Research. 2024;19(12):2661‐2672. doi:10.4103/1673-5374.391312 38595285 PMC11168510

[37] Shen T , Yue Y , Ba F , et al. Diffusion along perivascular spaces as marker for impairment of glymphatic system in Parkinson's disease. Parkinsons Dis. 2022;8(1):174. doi:10.1038/s41531-022-00437-1 PMC9772196 36543809

[38] Zhang S , Yu W , Zhang X , et al. Glymphatic dysfunction as a biomarker for post‐stroke cognitive impairment. Sci Rep. 2025;15(1):19382. doi:10.1038/s41598-025-04054-9 40461672 PMC12134357

[39] Wardlaw JM , Benveniste H , Nedergaard M , et al, colleagues from the Fondation Leducq Transatlantic Network of Excellence on the Role of the Perivascular Space in Cerebral Small Vessel Disease . Perivascular spaces in the brain: anatomy, physiology and pathology. Nat Rev Neurol. 2020;16(3):137‐153. doi:10.1038/s41582-020-0312-z 32094487

[40] Salvatore SV , Lambert PM , Benz A , et al. Periodic and aperiodic changes to cortical eeg in response to pharmacological manipulation. Journal of Neurophysiology. 2024;131(3):529‐540. doi:10.1152/jn.00445.2023 38323322 PMC11305649

[41] Kramer MA , Chu CJ . The 1/f‐like behavior of neural field spectra are a natural consequence of noise driven brain dynamics. Neuroscience. 2023. doi:10.1101/2023.03.10.532077. bioRxiv

[42] Kim K , Abramishvili D , Du S , et al. Meningeal lymphatics‐microglia axis regulates synaptic physiology. Cell. 2025;188(10):2705‐2719. doi:10.1016/j.cell.2025.02.022 40120575 PMC12086007

[43] Wen H , Liu Z . Broadband electrophysiological dynamics contribute to global resting‐state fMRI signal. J Neurosci. 2016;36(22):6030‐6040. doi:10.1523/JNEUROSCI.0187-16.2016 27251624 PMC4887567

[44] Jacob MS , Roach BJ , Sargent KS , Mathalon DH , Ford JM . Aperiodic measures of neural excitability are associated with anticorrelated hemodynamic networks at rest: a combined EEG‐fMRI study. NeuroImage. 2021;245:118705. doi:10.1016/j.neuroimage.2021.118705 34798229 PMC12799207

[45] Stitt I , Zhou ZC , Radtke‐Schuller S , Fröhlich F . Arousal dependent modulation of thalamo‐cortical functional interaction. Nat Commun. 2018;9(1):2455. doi:10.1038/s41467-018-04785-6 29941957 PMC6018110

[46] Lopez S , Hampel H , Chiesa PA , et al. The Association between posterior resting‐state EEG alpha rhythms and functional mri connectivity in older adults with subjective memory complaint. Neurobiology of Aging. 2024;137:62‐77. doi:10.1016/j.neurobiolaging.2024.02.008 38431999

[47] Lopez S , Del Percio C , Lizio R , et al. Patients with Alzheimer's Disease dementia show partially preserved parietal ‘hubs’ modeled from resting‐state alpha electroencephalographic rhythms. Front Aging Neurosci. 2023;15:780014. doi:10.3389/fnagi.2023.780014 36776437 PMC9908964

[48] Cao J , Li B , Li X . Identification of Alzheimer's disease brain networks based on EEG phase synchronization. BioMed Eng OnLine. 2025;24(1):32. doi:10.1186/s12938-025-01361-0 40059173 PMC11892187

[49] Chiarelli AM , Perpetuini D , Croce P , et al. Evidence of neurovascular un‐coupling in mild Alzheimer's disease through multimodal EEG‐fNIRS and multivariate analysis of resting‐state data. Biomedicines. 2021(4):337. doi:10.3390/biomedicines9040337 33810484 PMC8066873

[50] Ringstad G . Glymphatic imaging: a critical look at the DTI‐ALPS index. Neuroradiology. 2024;66(2):157‐160. doi:10.1007/s00234-023-03270-2 38197950

