# Supplementary material for: Glymphatic clearance as revealed by diffusion tensor imaging along the perivascular space (DTI‐ALPS) is associated with Alzheimer's disease neuropathology and periodic rsEEG alpha rhythms in mild cognitive impairment participants
Source: Alzheimers Dement (Amst). 2026 Jun 11;18(2):e70384. doi: 10.1002/dad2.70384 (PMC13254685; doi:10.1002/dad2.70384)
Supplement: Supplementary file 1 — Supporting Information [file DAD2-18-e70384-s002.docx]

***Supplementary Materials***

**Glymphatic clearance as revealed by diffusion tensor imaging along the perivascular space (DTI-ALPS) is associated with Alzheimer’s disease neuropathology and periodic rsEEG alpha rhythms in mild cognitive impairment participants**

Susanna Lopez^1^, Claudio Del Percio^1^, Roberta Lizio^1,2^, Giuseppe Noce^3^, Matteo Carpi^1^, Dario Arnaldi^4,5^, Francesco Famà^4,5^, Matteo Pardini^4,5^, Federico Massa^4,5^, Franco Marinozzi^6^, Fabiano Bini^6^, Giorgia Treves^1,6^, Andrea Soricelli^3,7^, Marco Salvatore^3^, Franco Giubilei^8^, Laura Ziccardi^8^, Bahar Güntekin^9,10^, Görsev Yener^11,12^, Raffaele Ferri^2^, Bartolo Lanuzza^2^, Fabrizio Stocchi^13,14^, Laura Vacca^13^, Chiara Coletti^13^, Francesco Infarinato^13^, Paola Romano^13^, Moira Marizzoni^15^, Giovanni B. Frisoni^16,17^, Paolo Barone^18^, Arianna Cappiello^18^, Sofia Cuoco^18^, Laura Bonanni^19^, Anita D’Anselmo^19^, Angelo Antonini^20,21^, Eleonora Fiorenzato^21^, Simone Cauzzo^21^, Roberta Biundo^21,22^, Fabrizia D’Antonio^23^, Maria Francesca De Pandis^24,25^, Simone Marziali^24^, Giuseppe Bruno^23^, Filippo Carducci^1,2$^& Claudio Babiloni^1,24$^

*1 Department of Physiology and Pharmacology "Vittorio Erspamer", Sapienza University of Rome, P.le Aldo Moro, 5, 00185, Rome, Italy;*

*2 Oasi Research Institute - IRCCS, Via Conte Ruggero, 73, 94018, Troina, Italy;*

*3 IRCCS Synlab SDN, Via Emanuele Gianturco, 113, 80143, Naples, Italy;*

*4 Dipartimento di Neuroscienze, Oftalmologia, Genetica, Riabilitazione e Scienze Materno-infantili (DiNOGMI), Università di Genova, Largo Paolo Daneo, 3, 16132, Genova, Italy;*

*5 IRCCS Ospedale Policlinico San Martino, Largo R. Benzi, 10, 16132, Genova, Italy;*

*6 Department of Mechanical and Aerospace Engineering, Sapienza University of Rome, Via Eudossiana, 18, 00184, Rome, Italy;*

*7 Department of Medical, Movement and Well-being Sciences, University of Naples Parthenope, Via Amm. F. Acton, 38, 80133, Naples, Italy;*

*8 Department of Neuroscience, Mental Health, and Sensory Organs, Sapienza University of Rome, Via di Grottarossa, 1035, 00189, Rome, Italy;*

*9 Department of Biophysics, School of Medicine, Istanbul Medipol University, Kavacık Mah. Ekinciler Cad. No: 19, Kavacık Kavşağı, 34810 Beykoz, İstanbul, Turkey;*

*10 Research Institute for Health Sciences and Technologies (SABITA), Neuroscience Research Center, Istanbul Medipol University, Kavacık Mah. Ekinciler Cad. No: 19, Kavacık Kavşağı, 34810 Beykoz, İstanbul, Turkey;*

*11 Department of Neurology, Faculty of Medicine, Dokuz Eylül University, 15 Temmuz Sağlık Sanat Yerleşkesi / İnciraltı 35340-İZMİR, İzmir, Türkiye;*

*12 IBG: International Biomedicine and Genome Center, 15 Temmuz Sağlık ve Sanat Kampüsü Mithatpaşa Cad. 58/5 35340, Balçova, İzmir, Turkey;*

*13 IRCCS San Raffaele Roma, Via della Pisana, 235, 00163, Rome, Italy;*

*14 San Raffaele Open University, Via di Val Cannuta, 247, 00166, Rome, Italy;*

*15 Biological Psychiatry Unit, IRCCS Istituto Centro San Giovanni di Dio Fatebenefratelli, Via Pilastroni, 4, 25125, Brescia, Italy;*

*16 Memory Center, Department of Rehabilitation and Geriatrics, University Hospitals and University of Geneva, Rue Gabrielle-Perret-Gentil 4 CH-1211, Genève 14, Genève, Switzerland;*

*17 Laboratory of Neuroimaging of Aging (LANVIE), University of Geneva, 24 rue du Général-Dufour 1211, Genève 4, Genève, Switzerland;*

*18 Department of Medicine, Surgery and Dentistry "Scuola Medica Salernitana", Neuroscience Section, University of Salerno, Via Salvador Allende, 43, 84081, Baronissi, Italy;*

*19 Department of Aging Medicine and Sciences, University “G. d’Annunzio” of Chieti-Pescara, Via dei Vestini, 31, 66100, Chieti, Italy;*

*20 IRCCS San Camillo Hospital, Via Alberoni, 70, 30126, Lido, Venice, Italy;*

*21 Parkinson and Movement Disorders Unit, Study Center for Neurodegeneration (CESNE), Department of Neuroscience, University of Padua, Via Giustiniani 2, 35128, Padova, Italy;*

*22 Department of General Psychology, University of Padua, Via Venezia, 8, 35131, Padua, Italy;*

*23 Department of Human Neurosciences, Sapienza University of Rome, P.le Aldo Moro, 5, 00185, Rome, Italy;*

*24 IRCCS San Raffaele Roma, Cassino site, Via Gaetano di Biasio, 1, 03043, Cassino, Italy;*

*25 Department of Human Science and Promotion of Quality of Life, San Raffaele Rome University, 00166, Rome Italy****.***

***$ = Equally contributing last authors***

**Corresponding Author:**

Prof. Claudio Babiloni

Department of Physiology and Pharmacology “V. Erspamer”

Sapienza University of Rome

P. le A. Moro 5, 00185, Rome, Italy

Phone: +39 0649910989. E-mail: [claudio.babiloni@uniroma1.it](mailto:claudio.babiloni@uniroma1.it)

**Running title:** DTI-ALPS, AD neuropathology and periodic alpha rsEEG rhythms

**Keywords**: Diffusion Tensor Imaging Along the Perivascular Space (DTI-ALPS); Alzheimer’s Disease (AD); Mild Cognitive Impairment (MCI); Resting-state Electroencephalographic (EEG) Rhythms; Spectral Parametrization; Periodic and aperiodic components of rsEEG power spectral density (PSD).

***Supplementary Methods***

*Participants*

The datasets for the present study were obtained from the international PharmaCog and the PDWAVES Consortium ([www.pdwaves.eu](http://www.pdwaves.eu)) archives. They included records from demographic-matched groups (i.e., groups with the same mean values of age, gender, and sex percentage), consisting of 53 ADMCI, 29 noADMCI, and 43 older cognitively unimpaired (Nold) participants. Those people were recruited from various clinical centers, including Sapienza University of Rome (Italy), Institute for Research and Evidence-based Care (IRCCS) “San Raffaele Roma” of Rome (Italy), IRCCS SDN of Naples (Italy), IRCCS Oasi Maria SS of Troina (Italy), IRCCS Ospedale Policlinico San Martino and DINOGMI (University of Genova, Italy), Hospital San Raffaele of Cassino (Italy), Hospital of University of Padua (Italy), Hospital of University of Chieti (Italy), Hospital of University of Salerno (Italy), Medipol University of Istanbul (Turkey), and Dokuz Eylül University of Izmir (Turkey).

Table Supplementary Material (SM)1 provides a summary of relevant demographic (i.e., age, sex, and education) and clinical (i.e., Mini Mental State Examination, MMSE, score) characteristics of the Nold, ADMCI and noADMCI groups, along with the results of the statistical analyses computed to determinate the presence or absence of statistically significant differences between these groups in terms of age (ANOVA), sex (Fisher test), education (ANOVA), and clinical markers (Kruskal-Wallis test for MMSE). There were no statistically significant differences in age, sex, and education between the three groups (p > 0.05), whereas Nold participants had higher MMSE scores than ADMCI and noADMCI participants (p < 0.05). There were no statistically significant differences in age, sex, education, or MMSE score between the ADMCI and noADMCI groups (p > 0.05).

|  | **Nold** | **ADMCI** | **noADMCI** | **Statistical analysis** |
| --- | --- | --- | --- | --- |
| **N** | 43 | 53 | 29 | - |
| **Age  (mean in years ± SE)** | 68.9 ± 1.4 | 70.5 ± 0.9 | 69.0 ± 1.4 | ANOVA:  p = n.s. |
| **Sex  (F/M, %)** | 26/17, 39% | 29/24, 45% | 20/9, 31% | χ^2^ test:  p = n.s. |
| **Education  (mean in years ± SE)** | 11.3 ± 0.7 | 11.3 ± 0.6 | 10.1 ± 0.8 | ANOVA:  p = n.s. |
| **MMSE  (mean score ± SE)** | 28.0 ± 0.2 | 25.1 ± 0.2 | 25.5 ± 0.4 | Kruskal-Wallis:  p < 0.05  **Nold > ADMCI, noADMCI** |

**Table Supplementary Materials (SM)1.** Mean values (± standard error mean, SE) of the demographic and clinical (MMSE) data, together with the results of their statistical comparisons (p < 0.05) in the groups of participants with mild cognitive impairment due (ADMCI) or not due (noADMCI) to Alzheimer’s disease neuropathology assessed through cerebrospinal fluid (CSF) biomarkers. *Legend: AD = Alzheimer’s disease; ADMCI = mild cognitive impairment due to AD; noADMCI = mild cognitive impairment not due to AD; MMSE = Mini-Mental State Examination; M/F = males/females; n.s. = not significant (p > 0.05).*

*Diagnostic criteria*

The clinical inclusion criteria for both ADMCI and noADMCI participants are reported in^1^ and were as follows: (1) age range of 55 to 90 years; (2) self-reported memory concerns by the participant; (3) Mini-Mental State Examination (MMSE) score of 24 or higher; (4) Clinical Dementia Rating (CDR) score of 0.5; (5) Logical memory test performance of 1.5 standard deviations (SD) below the age-adjusted mean, indicating cognitive impairment that does not significantly affect functional independence in daily activities; (6) Geriatric Depression Scale (15-item GDS) score of 5 or lower; (7) Modified Hachinski Ischemia score of 4 or lower; (8) at least 5 years of education; and (9) diagnosis of single or multi-domain MCI.

The clinical exclusion criteria for both ADMCI and noADMCI groups included: (1) mixed dementia; (2) chronic use of neuroleptics, narcotics, analgesics, sedatives, or hypnotics (e.g., benzodiazepines); (3) ongoing participation in a clinical trial involving disease-modifying drugs; (4) diagnosis of major psychiatric disorders (i.e., depression, etc.) or neurological illness not related to cognitive deficits; (5) diagnosis of epilepsy or report of seizures or epileptiform EEG activity in the past; (6) use of antiepileptics; and (7) chronic use of neuroleptics, narcotics, analgesics, sedatives or hypnotics.

The clinical status of ADMCI was determined based on positivity for CSF core AD markers, as reported below, and a compatible neurodegenerative pattern on structural MRI or FDG-PET^2^. All noADMCI subjects, conversely, were CSF negative.

Both ADMCI and noADMCI participants underwent comprehensive cognitive assessment^1^, which included the following: (1) global cognitive function (MMSE score); (2) episodic memory evaluated with immediate and delayed recall tasks of Logical Memory and the Rey Auditory Verbal Learning Test; (3) executive functions and attention assessed using the Trail Making Test (TMT) parts A and B; (4) language abilities measured using the 1-minute Verbal Fluency Test for letters and categories; (5) planning abilities and visuospatial skills evaluated with the Clock Drawing and Copy Test.

All Nold participants underwent an interview and cognitive screening (including MMSE and GDS), as well as physical and neurological examinations, to exclude subjective memory complaints (SMC), cognitive deficits, and mood disorders. All Nold participants had an MMSE score ≥ 27, a CDR score of 0, and a GDS score below the threshold of 5 (no depression) or were assessed as having no depression after an interview with a physician or a clinical psychologist at the time of enrollment. Nold participants with a history of past or present neurological or psychiatric illness were also excluded. In addition, Nold participants affected by any chronic systemic disease (e.g., diabetes mellitus) were excluded, as were Nold participants who were chronically taking psychoactive drugs. Unfortunately, structural magnetic resonance imaging (sMRI), cerebrospinal fluid (CSF), and APOE genotyping were not available for the present Nold participants.

*Magnetic Resonance Imaging (MRI) data acquisition and preparation*

All MRI scans were acquired on 3.0 Tesla scanners across multiple sites using systems from General Electric, Philips, and Siemens, in accordance with harmonized acquisition protocols as part of a more extended project reported as IMI WP5 PharmaCog (also referred to as the European ADNI study^3^. The MRI protocol included T1-weighted, fluid-attenuated inversion recovery (FLAIR), and diffusion tensor imaging (DTI) sequences according to the ADNI-2 protocol (<http://adni.loni.usc.edu/methods/documents/mri-protocols>). MRI acquisition procedures included both a multisite qualification phase, aimed at ensuring data quality and inter-site comparability, and a participant scanning phase. Quality assurance procedures comprised regular phantom scans and site-specific checks to ensure scanner stability and protocol adherence across centers.

During the participant scanning phase, all imaging data were anonymized at each site and transferred to a centralized database for processing. Prior to analysis, all images underwent visual quality control to exclude scans with major artifacts, including motion, wrap-around, radiofrequency interference, signal intensity inhomogeneities, or incomplete brain coverage. When multiple T1-weighted images were acquired within the same session, they were averaged to improve signal-to-noise ratio prior to further processing. Scans requiring substantial manual intervention for processing or presenting significant quality issues were excluded from subsequent analyses. Details of the scanners and sequences used at each site have been previously reported^3^. Diffusion tensor imaging (DTI) data were acquired using a spin-echo echo-planar imaging (SE-EPI) sequence with the following parameters: repetition time (TR) = 9300 ms, echo time (TE) = 84 ms, flip angle = 90°, voxel size = 2.0 × 2.0 × 2.0 mm³, matrix size = 112 × 112, field of view = 240 × 240 mm², and 64 axial slices per volume. A total of 36 volumes were acquired, including 30 diffusion-weighted volumes with a b value of 700 s/mm² and 6 non-diffusion-weighted (b0) volumes.

*Volumetric and cortical thickness markers*

Structural T1-weighted images were processed as previously described. When multiple T1 acquisitions were available within the same session, images were averaged to improve signal-to-noise ratio. The resulting images were processed using FreeSurfer v5.1.0^4,5^ with the standard automated pipeline (*recon-all*), including skull stripping, intensity normalization, segmentation of gray and white matter, and cortical surface reconstruction. Cortical parcellation was performed according to the Desikan–Killiany atlas. From the FreeSurfer outputs, subject-specific estimates of cortical thickness and subcortical volumes were obtained. In the present study, we focused on a subset of regions of interest (ROIs) known to be relevant for AD, including the hippocampus and amygdala for volumetric measures, and mesial temporal (parahippocampal gyrus, fusiform gyrus, entorhinal cortex) and parietal regions (precuneus and cuneus) for cortical thickness. In addition, control regions not expected to be primarily affected by the pathology were included, namely the cerebellar cortex for volumetric measures and the precentral gyrus for cortical thickness. For each structure, left and right hemisphere measures were extracted separately. Volumetric measures were normalized to total intracranial volume to account for inter-individual differences in head size. All segmentation outputs were visually inspected for quality assurance prior to statistical analyses to confirm the absence of major errors. No manual editing of the segmentations was performed.

*White matter abnormalities*

White matter abnormalities were assessed using two complementary approaches based on different MRI sequences. First, white matter lesions (WML) were evaluated on FLAIR images using the Age-Related White Matter Changes (ARWMC) rating scale. Lesions were visually rated in frontal, parieto-occipital, temporal, infratentorial regions, and basal ganglia, with total scores ranging from 0 to 30, where higher scores indicate greater cerebrovascular burden. Second, white matter hypointensities were derived from T1-weighted images using the automated segmentation implemented in FreeSurfer (version 5.1.0; aseg.mgz output). These regions correspond to voxels within the white matter exhibiting lower signal intensity relative to surrounding tissue on T1-weighted images. The total volume of white matter hypointensities was extracted for each participant and normalized to total intracranial volume. While FLAIR-based measures are considered the reference standard for detecting white matter lesions, T1-derived white matter hypointensities provide a fully automated and standardized estimate of white matter signal abnormalities^6^. The two measures were therefore treated as complementary indices of white matter damage.

*Diffusion Tensor Imaging (DTI) processing*

All DTI images were processed by two experts using a double-blind coprocessing method according to uniform criteria. DTI data were preprocessed using the MRtrix3 software package (version 3.0.3; [www.mrtrix.org](http://www.mrtrix.org))^7^ in combination with the FMRIB Software Library (FSL, release 6.0.7; <https://fsl.fmrib.ox.ac.uk/fsl/>)^8^. Raw DTI data were converted into 4D NIfTI format using the dcm2niix software (https://pypi.org/project/dcm2niix/)^9^. Converted imaging data were first denoised using the Marchenko–Pastur principal component analysis (MP-PCA)^10^ approach (*dwidenoise*) and corrected for Gibbs ringing artifacts (*mrdegibbs*)^11^, both implemented in MRtrix3. Residual eddy current–induced distortions and subject motion were then corrected using FSL’s GPU-accelerated EDDY tool (*eddy_cuda*)^12^, which estimates eddy current–related field inhomogeneities and head movement in a single optimization framework and applies them to realign all diffusion-weighted volumes to a common reference. After motion and distortion correction, brain extraction was performed on the mean b0 image using FSL BET, and diffusion tensors were fitted voxel-wise using FSL (*dtifit*) to obtain fractional anisotropy (FA) and directional diffusivity maps along the right–left (D_xx_), anterior–posterior (D_yy_), and inferior–superior (D_zz_) axes.

For spatial normalization, individual T1-weighted structural images were first skull-stripped using FSL BET and then registered to the MNI152_T1_1mm_brain template using FSL registration tools. The resulting transformations were subsequently applied to the diffusion tensor–derived maps using FSL applywarp, bringing D_xx_, D_yy_, and D_zz_ into MNI template space while preserving their orientation for subsequent ALPS calculations. For each subject, the results of eddy current and motion correction, brain extraction, and spatial normalization were visually inspected to ensure the accuracy of the preprocessing steps.

*Diffusion Tensor Imaging (DTI) processing*

All DTI images were processed by two experts using a double-blind coprocessing method according to uniform criteria. DTI data were preprocessed using the MRtrix3 software package (version 3.0.3; [www.mrtrix.org](http://www.mrtrix.org))^7^ in combination with the FMRIB Software Library (FSL, release 6.0.7; <https://fsl.fmrib.ox.ac.uk/fsl/>)^8^. Raw DTI data were converted into 4D NIfTI format using the dcm2niix software (https://pypi.org/project/dcm2niix/)^9^. Converted imaging data were first denoised using the Marchenko–Pastur principal component analysis (MP-PCA)^10^ approach (*dwidenoise*) and corrected for Gibbs ringing artifacts (*mrdegibbs*)^11^, both implemented in MRtrix3. Residual eddy current–induced distortions and subject motion were then corrected using FSL’s GPU-accelerated EDDY tool (*eddy_cuda*)^12^, which estimates eddy current–related field inhomogeneities and head movement in a single optimization framework and applies them to realign all diffusion-weighted volumes to a common reference. After motion and distortion correction, brain extraction was performed on the mean b0 image using FSL BET, and diffusion tensors were fitted voxel-wise using FSL (*dtifit*) to obtain fractional anisotropy (FA) and directional diffusivity maps along the right–left (D_xx_), anterior–posterior (D_yy_), and inferior–superior (D_zz_) axes.

For spatial normalization, individual T1-weighted structural images were first skull-stripped using FSL BET and then registered to the MNI152_T1_1mm_brain template using FSL registration tools. The resulting transformations were subsequently applied to the diffusion tensor–derived maps using FSL applywarp, bringing D_xx_, D_yy_, and D_zz_ into MNI template space while preserving their orientation for subsequent ALPS calculations. For each subject, the results of eddy current and motion correction, brain extraction, and spatial normalization were visually inspected to ensure the accuracy of the preprocessing steps.

*Diffusion tensor imaging along the perivascular space (DTI-ALPS) calculation*

The DTI-ALPS index provides an indirect measure of water diffusivity along perivascular spaces (PVS) within major white matter tracts that run orthogonal to the lateral ventricles, in particular the superior corona radiata (SCR) and superior longitudinal fasciculus (SLF). In these regions, medullary veins and their surrounding PVS are predominantly oriented along the left–right (x) axis. The index leverages this geometry by using diffusivity along the x-axis in SCR and SLF as a surrogate for perivascular flow. In contrast, diffusivity along the y-axis in the SCR and along the z-axis in the SLF, directions approximately perpendicular to both the fiber orientation and the PVS, is used to estimate background tissue diffusivity, which is less related to glymphatic transport.

Spherical regions of interest (ROIs) with a 5 mm diameter were placed bilaterally in the SCR and SLF, using previously published coordinates^13,14^ as follows: left SCR (116, 110, 99), left SLF (128, 110, 99), right SCR (64, 110, 99), and right SLF (52, 110, 99), defined in MNI152_T1_1mm template voxel space, following the original description by Taoka and colleagues^15^. Figure Supplementary Materials (SM) 1 illustrates the placement of the ROIs.


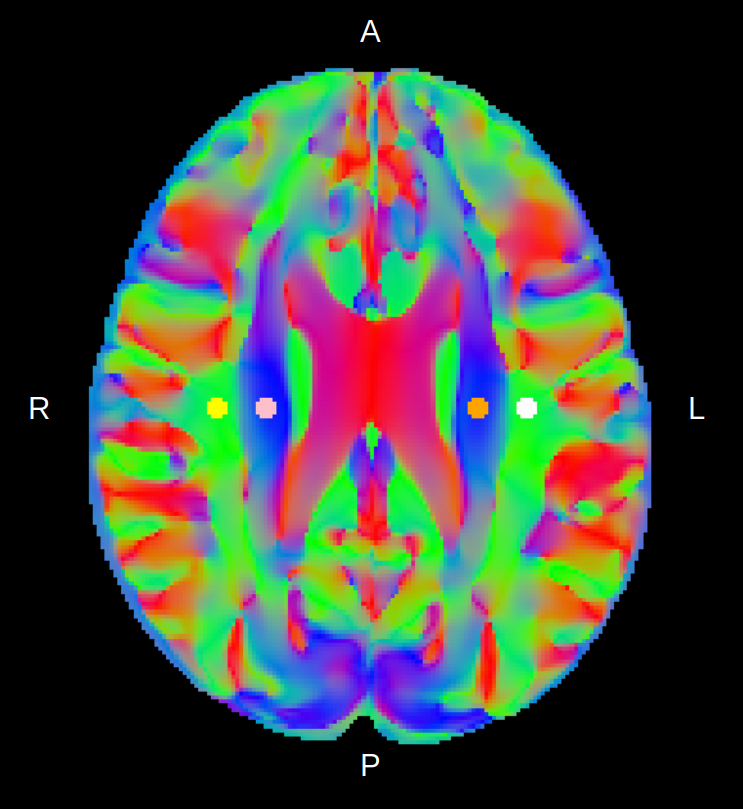


**Figure Supplementary Materials (SM) 1.***Regions of interest (ROIs) for the DTI-ALPS index calculation.* Axial color-coded diffusion tensor imaging (DTI) map in MNI152 standard space showing the placement of spherical regions of interest (ROIs) used for DTI-ALPS index calculation. ROIs (5 mm diameter) were positioned bilaterally within the superior corona radiata (SCR) and superior longitudinal fasciculus (SLF). The color encoding follows the conventional DTI orientation scheme, with red indicating left–right (x-axis), green anterior–posterior (y-axis), and blue superior–inferior (z-axis) diffusion directions. The selected ROIs sample diffusivity along the x-axis, corresponding to the predominant orientation of perivascular spaces adjacent to medullary veins, whereas diffusivities along the y-axis in the SCR and along the z-axis in the SLF provide reference measures of diffusion perpendicular to both the dominant fiber orientation and the perivascular spaces for ALPS index computation provide reference measures of diffusivity perpendicular to the perivascular spaces and the main white matter fiber orientation, thereby serving as normalization terms in the ALPS index computation.

Specifically, the DTI-ALPS index was computed as:

$DTI-ALPS= \frac{mean (D_{xx, proj}, D_{xx,assoc})}{mean (D_{yy,proj}, D_{zz,assoc})}$ (1)

Where D_xx,proj_ is the *x*-axis DTI diffusivity in the area of projection fiber, D_xx,assoc_ is the *x*-axis DTI diffusivity in the area of association fibers, D_yy,proj_ is the *y*-axis DTI diffusivity in the area of projection fiber, and D_zz,assoc_ is the *z*-axis DTI diffusivity in the area of association fibers. A high DTI-ALPS index indicates that the diffusion of free water in the *x*-direction, or Brownian motion, is dominant. In contrast, a small DTI-ALPS index indicates that free water movement in the *x*-direction is not dominant, consistent with reduced perivascular water movement and impaired glymphatic-mediated clearance. The average of the left and right DTI-ALPS indices (mean DTI-ALPS index) was then calculated. A DTI-ALPS index closes to 1.0 reflects minimal diffusivity, whereas a higher value indicates greater diffusivity. All resource-heavy computation steps were performed on the computing infrastructure at Prof. Carducci’s lab.

*rsEEG recordings*

The rsEEG recordings were conducted using local routine professional digital EEG systems licensed for clinical applications. All rsEEG recordings were performed in the morning to minimize circadian rhythm-related variations. Standard instructions for the resting-state condition emphasized staying awake, psychophysically relaxed, with mind wandering, and following the experimenter’s requests to keep the eyes closed and open during the rsEEG recording. The experiments monitored the participant’s behavioral state during EEG recordings and annotated any deviations and alarms.

A common electrode montage of 19 scalp exploring electrodes, placed according to the 10–20 system (i.e., O1, O2, P3, Pz, P4, T3, T5, T4, T6, C3, Cz, C4, F7, F3, Fz, F4, F8, Fp1, and Fp2), characterized the rsEEG data recordings in all clinical units and was used for the data analysis. The reference electrode was typically placed between Fz and Cz of the 10-20 system, and the ground electrode was in the posterior midline. To minimize the influence of differences in electrode placement, all EEG data were re-referenced to the common average for data analysis.

Electrooculographic (EOG) activity, recorded with a standard bipolar montage, was also recorded to monitor and control eye movements and blinking.

As minimum standards across all clinical units, electrophysiological data were acquired with a bandpass of 0.03–70 Hz and a minimum sampling frequency of 256 Hz. This acquisition configuration preserved slow EEG fluctuations while avoiding excessive high-pass filtering during signal acquisition and provided a sufficient buffer against aliasing while preserving high-frequency EEG activity.

*Preliminary rsEEG data analysis*

The rsEEG data were centrally analyzed by experts at Sapienza University of Rome, who were blinded to the participant’s diagnosis, in line with a previous investigation of our Workgroup1. The recorded rsEEG data were exported in either European data format (.edf) or EEGLAB set (.set) files and subsequently processed offline using the EEGLAB toolbox (Delorme and Makeig, 2004; version eeglab14_1_2b) running in the MATLAB software (Mathworks, Natick, MA, USA; version: R2014b).

To harmonize the data, all artifact-free rsEEG datasets were filtered offline in the 0.1–45 Hz band and, when necessary, down-sampled to a uniform sampling frequency of 256 Hz. Resampling was applied only after low-pass filtering to avoid aliasing effects. The initial 0.03–70 Hz bandpass referred exclusively to acquisition settings of the clinical EEG systems, whereas all spectral analyses were performed on uniformly preprocessed data filtered offline between 0.1 and 45 Hz.

For preprocessing, the rsEEG data were segmented into 2-second epochs (i.e., 5 minutes of data corresponded to 150 epochs of 2 seconds each) and analyzed offline. A three-step procedure was implemented to detect and remove the following items: (1) recording channels (electrodes) showing prolonged artifactual rsEEG activity due to bad electric contacts or other reasons; (2) rsEEG epochs containing artifacts from channels that generally had good signals; and (3) intrinsic components of the rsEEG epochs affected by artifacts.

The initial step involved a visual examination of rsEEG activity by two independent experimenters from a panel of four experts (i.e., S.L., C.D.P., R.L., and G.N.) to identify electrodes with irreparable artifacts, typically removing no more than three per participant. If a clinical unit used a digital rsEEG system with more than 19 electrodes, the removed electrodes were replaced with the nearest non-selected electrodes. These additional electrodes, along with artifact-free ones, were used to interpolate data at the locations of the removed electrodes, ensuring all participants had artifact-free rsEEG data.

In the second step, the same experimenters visually reviewed the rsEEG epochs to identify and eliminate those contaminated by muscular, ocular, head movements, or non-physiological artifacts. Muscle tension artifacts were detected by examining power density spectra, which revealed unusually high values in the 30-70 Hz range, deviating from the typical decline in power density.

The third step involved applying independent component analysis (ICA) in the EEGLAB toolbox to remove components corresponding to residual artifacts, including eye movements, involuntary head movements, neck and shoulder muscle tension, and electrocardiographic activity. Fewer than 3 ICA components were removed from each dataset, which were then reconstructed using the remaining artifact-free ICA components. To ensure data integrity, the presumed artifact-free rsEEG epochs underwent a visual double-check by independent experimenters, who confirmed their inclusion or exclusion.

The final artifact-free rsEEG epochs were re-referenced to the common average reference. After these procedures, the artifact-free epochs maintained a similar proportion (over 75%) of the total rsEEG activity recorded across all participants. More specifically, the average number of artifact-free epochs were the following: Nold = 98 ± 3.73, ADMCI = 121 ± 3.34, noADMCI = 129 ± 4.29.

*rsEEG power spectral density calculation*

Power spectral density (PSD) was computed from artifact-free rsEEG epochs for each participant. The Power spectral density (PSD) was computed from artifact-free rsEEG epochs for each participant. The PSD analysis was performed using the pop_spectopo function from the EEGLAB toolbox. A window size equal to the sampling rate (i.e., 1-second Hanning window without overlap) was applied, resulting in a frequency resolution of 1 Hz. The absolute PSD values were obtained for frequency bins ranging from 1 to 40 Hz and used as input for the subsequent analysis of the periodic (PSD peaks) and aperiodic components derived from the EEG activity. Spectral analyses were restricted to the 1–40 Hz range, well below the Nyquist frequency associated with the final sampling rate.

The PSD values were processed using FOOOF, implemented via the EEGLAB plugin *specparam* (<https://github.com/bfbarry/EEGLAB-specparam/>). We used the following settings validated in a previous reference study in ADD patients^16^: fixed mode (no “knee”), PSD peak width limits from 1 to 12 Hz, a maximum of 7 PSD peaks, a minimum PSD peak magnitude of 0 (arbitrary units), and a PSD peak “elevation” threshold of 1 (arbitrary units). With these settings, FOOOF decomposed the spectrum of PSD values from 1 to 40 Hz into a broadband aperiodic component superimposed on periodic PSD peaks^17^. In other words, this decomposition allowed for the dissociation of the aperiodic (non-oscillatory) and periodic (oscillatory) components from the raw EEG PSD values in the spectrum at each electrode^17^. For each electrode, the aperiodic component was modeled as a 1/f^χ^ function in the log-log space, where *χ* corresponds to the aperiodic exponent (i.e., the negative slope) computed over the PSD values from 1 to 40 Hz in the spectrum. Exponent values were obtained for each electrode, along with offset values, representing the intercepts of the aperiodic component in the log-log space. At the same time, the periodic components for each electrode were modeled as Gaussian PSD peaks rising above the background of the aperiodic component^18^. To obtain estimates of the aperiodic-corrected PSD, the modeled aperiodic PSD component was subtracted from the raw log-transformed rsEEG PSD spectrum at 1–40 Hz. The aperiodic-corrected PSD peak values were expressed in arbitrary units.

Oscillatory peaks were identified as deviations from the aperiodic component and modeled using Gaussian functions, providing estimates of center frequency, power, and bandwidth. As peak parameters were derived from continuous parametric fits, they were not constrained by the discrete frequency sampling of the PSD. This approach allows sub-bin frequency precision despite discrete spectral sampling. The individual alpha frequency peak (IAFp^18^) was defined as the center frequency of the most prominent oscillatory peak within 5–15 Hz at posterior electrodes (P3, Pz, P4, P7, P8, O1, O2), as identified by the spectral parameterization procedure applied to the aperiodic-corrected spectrum. Corrected PSDs at the IAFp were computed for each electrode and retained for subsequent analyses. An extended alpha search range (5–15 Hz) was adopted to account for inter-individual variability in alpha peak frequency, particularly in aging populations.

The delta (1-3 Hz) and theta (4-7 Hz) frequency bands were defined based on standard fixed frequency ranges from reference rsEEG studies^19^. The alpha band was defined based on the IAFp and further divided into sub-bands, namely the low (from IAFp-2 Hz to IAFp; 5 Hz as the lower limit) and the high (from IAFp to IAFp + 2 Hz; 13 Hz as the upper limit) alpha bands. This choice was made because, in the rsEEG data, dominant low-frequency alpha rhythms (alpha 1 and alpha 2) may reflect the synchronization of diffuse cortical neural networks that regulate fluctuations in the subject's global wakefulness and vigilance states. In contrast, the high-frequency alpha rhythms (alpha 3) may denote the (de)synchronization of more selective cortical neural networks specialized in processing modal-specific or semantic information during event-related paradigms^18^. We focused on the individual rsEEG alpha frequency bands because a mean slowing in the peak frequency of the alpha power density may characterize a clinical group without any substantial change in the magnitude of the power density. In that case, using fixed-frequency bands would result in a statistical artifact erroneously showing rsEEG alpha power density values lower in the clinical than in the control group. To avoid the inclusion of spurious frequencies typically not within the alpha range we limited the lower and upper limit of the alpha sub-bands, as mentioned above.

To capture both global and regional topographic features of aperiodic and periodic component of rsEEG PSD components, we averaged the aperiodic (exponent and offset) and periodic (delta, theta, low alpha, high alpha) rsEEG PSD variables across all the electrodes (global values) and in the following scalp regions of interest: frontal (Fp1, Fp2, F3, Fz, F4, F7, F8), central (T7, T8, C3, Cz, C4), and posterior (P3, Pz, P4, P7, P8, O1, O2).

*Main statistical analysis*

Descriptive statistics were reported as means and standard errors of the mean for continuous variables, and as counts and percentages for categorical variables. Between-group comparisons for demographic, clinical, genetic, neuropathological, and neuropsychological measures were performed using independent-samples T tests (for ADMCI vs noADMCI comparisons) and ANOVAs (for ADMCI vs noADMCI vs Nold comparisons) for normally-distributed variables (age, education, AD neuropathology MRI markers, neuropsychological scores) or Mann-Whitney U tests (for ADMCI vs noADMCI comparisons) and Kruskal-Wallis (for ADMCI vs noADMCI vs Nold comparisons) for MMSE score as non-interval variable. χ^2^ test was used for categorical data (i.e., sex and APOE genotyping). P-values below 0.05 were considered statistically significant with FDR multiple comparisons correction.

Associations between DTI-ALPS, rsEEG variables, CSF biomarkers, WML, and the episodic memory/executive functions (Logical Memory immediate recall, Logical Memory Test delayed recall, RAVLT immediate recall, RAVLT delayed recall) were assessed using general linear models (linear model fit by Ordinary Least Squares, OLS) including Group and Group×predictor interactions. Significant interactions were further explored using simple effects analyses with FDR correction. Variables violating normality assumptions were log-transformed. Outliers were evaluated using an iterative Grubbs’ test (p < 0.001). Statistical analyses were conducted using Jamovi (v2.5.6) and MATLAB (R2024b).

Due to the factorial design of the present study, we used ANOVA and a general linear regression model to compare the periodic and aperiodic components of the rsEEG PSD and the DTI-ALPS index between the ADMCI and noADMCI groups. However, using these models assumes that the dependent variables approximate Gaussian distributions. To tackle this issue, the Shapiro-Wilk and Kolmogorov-Smirnov tests were used to determine whether the distributions of the variables in each ANOVA or general linear regression model approximated Gaussian distributions (the null hypothesis of non-Gaussian distributions was evaluated at p < 0.05). If this were not the case, the variable distributions were processed by the Log10 transformation and re-tested. Such a transformation is a popular method for converting skewed distributions with all positive values (as periodic rsEEG PSD values are) into Gaussian distributions, thereby improving the reliability of ANOVA and general linear regression results. The procedure approximated all periodic rsEEG PSD distributions to Gaussian distributions, allowing the use of ANOVA and general linear regression models. The same procedure was applied for the CSF AD biomarkers, cortical thickness, and white matter (WM) T2-lesions and T1-hypointensities to achieve normal distribution. The other variable distributions already satisfied the assumption of normality (p > 0.05; no need for Log10 transformation). All variable distributions were also visually checked using QQ plots. For the ANOVA design, the degrees of freedom were corrected by the Greenhouse-Geisser procedure when appropriate.

Four statistical sessions were conducted to evaluate the working study hypotheses. The freeware Jamovi (version 2.5.6) with the GAMLj3 module, MATLAB (version R2024b; MathWorks, Natick, MA, USA), and the Visual Studio Code platform were used for computational procedures, statistical analyses, and plotting.

The first statistical session was performed to evaluate the hypothesis that the DTI-ALPS index might differ (1) between the ADMCI and noADMCI groups, and (2) between the left and right ROI (p < 0.05). To this aim, an ANOVA was used with the DTI-ALPS index as the dependent variable. The ANOVA factors were Group (ADMCI, noADMCI) and ROI (left, right). The Duncan test was used for planned post-hoc comparisons between the ADMCI and noADMCI groups (p < 0.05, FDR-corrected).

The second statistical session was performed to evaluate the hypothesis that the periodic and aperiodic components of the rsEEG PSD might differ (1) between the Nold, ADMCI, and noADMCI groups, and (2) among the scalp regions of interest (frontal, central, and posterior). Several ANOVA designs evaluated differences in the periodic and aperiodic components of the rsEEG PSD spectra among the three groups (Nold, ADMCI, and noADMCI) across three regions of interest (ROIs: frontal, central, and parieto-occipital). For the aperiodic components of the rsEEG PSD, the exponent and offset were used as dependent variables (one variable per model), with Group (Nold, ADMCI, and noADMCI) and ROI (frontal, central, parieto-occipital) as factors. For the periodic components of the rsEEG PSD, the rsEEG PSD were used as dependent variables, with Group (Nold, ADMCI, and noADMCI), Band (delta, theta, low alpha, high alpha), and ROI (frontal, central, parieto-occipital) as factors. The Duncan test was used for planned post-hoc comparisons between the ADMCI and noADMCI groups (p < 0.05, FDR-corrected).

The third statistical session was performed to evaluate a more specific hypothesis that the periodic and aperiodic components of the rsEEG PSD might differ (1) between the ADMCI and noADMCI groups, and (2) among the scalp regions of interest (frontal, central, and posterior). Several ANOVA designs evaluated differences in the periodic and aperiodic components of the rsEEG PSD spectra between the two groups (ADMCI, noADMCI) across three regions of interest (ROIs: frontal, central, and parieto-occipital). For the aperiodic components of the rsEEG PSD, the exponent and offset were used as dependent variables (one variable per model), with Group (ADMCI, noADMCI) and ROI (frontal, central, parieto-occipital) as factors. For the periodic components of the rsEEG PSD, the rsEEG PSD were used as dependent variables with the Group (ADMCI, noADMCI), Band (delta, theta, low alpha, high alpha), and ROI (frontal, central, parieto-occipital ) as factors. The Duncan test was used for planned post-hoc comparisons between the ADMCI and noADMCI groups (p < 0.05, FDR-corrected).

The fourth statistical session evaluated the working hypothesis that the DTI-ALPS index may be associated with the white matter (WM) hypointensities, the Aβ1−42, p-tau and Aβ1−42/p-tau neuropathology, the periodic and aperiodic components of the rsEEG PSD, and the episodic memory/executive functions (measured by Logic Memory and Rey Auditory Verbal Learning task immediate and delayed recall scores), in relation to the two groups (ADMCI, noADMCI). Several general linear models (GLMs; p < 0.05) were implemented, one for each target/predictor variable. In particular, the DTI-ALPS index was considered a predictor to evaluate its effects on the periodic and aperiodic components of the rsEEG PSD and on memory/executive functions (target). On the other hand, the DTI-ALPS index was considered the target variable when evaluating the effects of WM hypointensities or Aβ1-42/A/p-tau neuropathology. The Group (ADMCI, noADMCI) and the 2-way interaction between the Group and the predictor variable in each model were considered as predictors in the GLR (p < 0.05). Post-hoc analysis focused on the comparison between the ADMCI and noADMCI groups (p < 0.05, FDR-corrected).

The potential impact of any outliers in the distribution of the variables, as mentioned earlier, on the statistical results was evaluated using the iterative (leave-one-out) Grubbs’ test to detect outliers. The null hypothesis of non-outlier status was evaluated at a threshold of p > 0.001 to remove individual values with a high probability of being outliers.

***Supplementary Materials Results***

*DTI-ALPS index in noADMCI and ADMCI participants*

Figure SM2 illustrates the mean values (± standard error mean, SE) of the left and right DTI-ALPS index. A statistically significant 2-way ANOVA interaction (F(1, 80) = 5.75; p = 0.019) between the Group (ADMCI, noADMCI) and ROI (left, right) factors was observed.

ADMCI group was characterized by lower left and right DTI-ALPS indexes (p = 0.038 and p = 0.004, respectively, Duncan post-hoc FDR-corrected) as compared to the noADMCI group. In the following statistical analysis, we considered the average between left and right DTI-ALPS index in relation to the other global variables (WML, CSF AD neuropathology, rsEEG PSD, and cognition) considered in the present study.

*
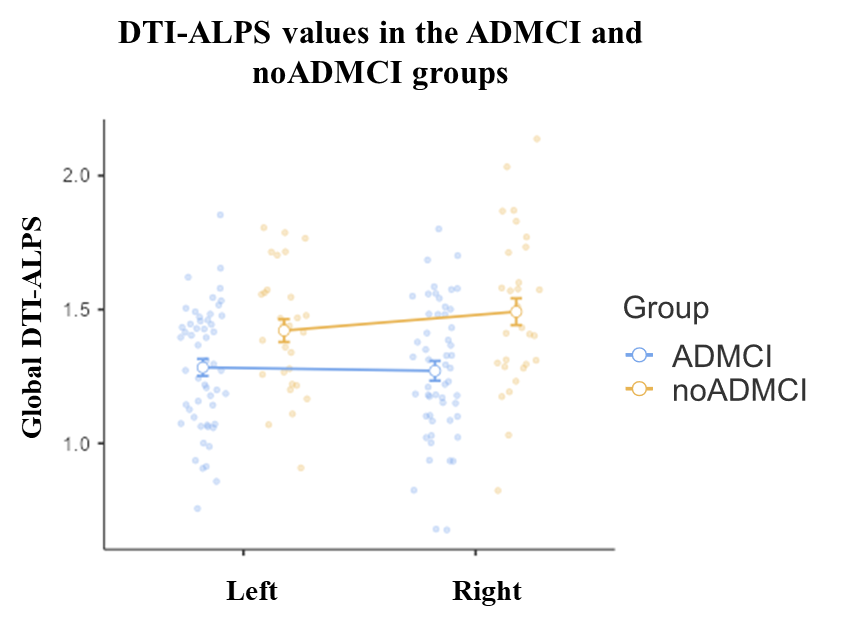
*

**Figure SM2.** *Mean values (± standard error mean, SE) of the left and right DTI-ALPS index.* A statistically significant 2-way ANOVA interaction (F(1, 80) = 5.75; p = 0.019) between the Group (ADMCI, noADMCI), and ROI (Left, Right) factors unveiled that the ADMCI group was characterized by lower left and right DTI-ALPS indexes (p = 0.038 and p = 0.004, respectively, Duncan post-hoc FDR-corrected) as compared to the noADMCI group. *Legend: AD = Alzheimer’s disease; ADMCI = mild cognitive impairment due to AD; noADMCI = mild cognitive impairment not due to AD; DTI-ALPS = diffusion tensor imaging along the perivascular space.*

*Association between Aβ1−42 and p-tau neuropathology and global DTI-ALPS*

Figure SM3 illustrates the association between the Group and Aβ1−42 or p-tau neuropathology (as predictors) and the global DTI-ALPS index (target variable) in the noADMCI and ADMCI groups. No statistically significant main (Aβ1-42 or p-tau) or interacting effect (Group X Aβ1-42 or p-tau) was observed including the Aβ1-42 (p = 0.607 and p = 0.410, respectively) or the p-tau (p = 0.629 and p = 0.095, respectively) and the Group factors was observed.

*
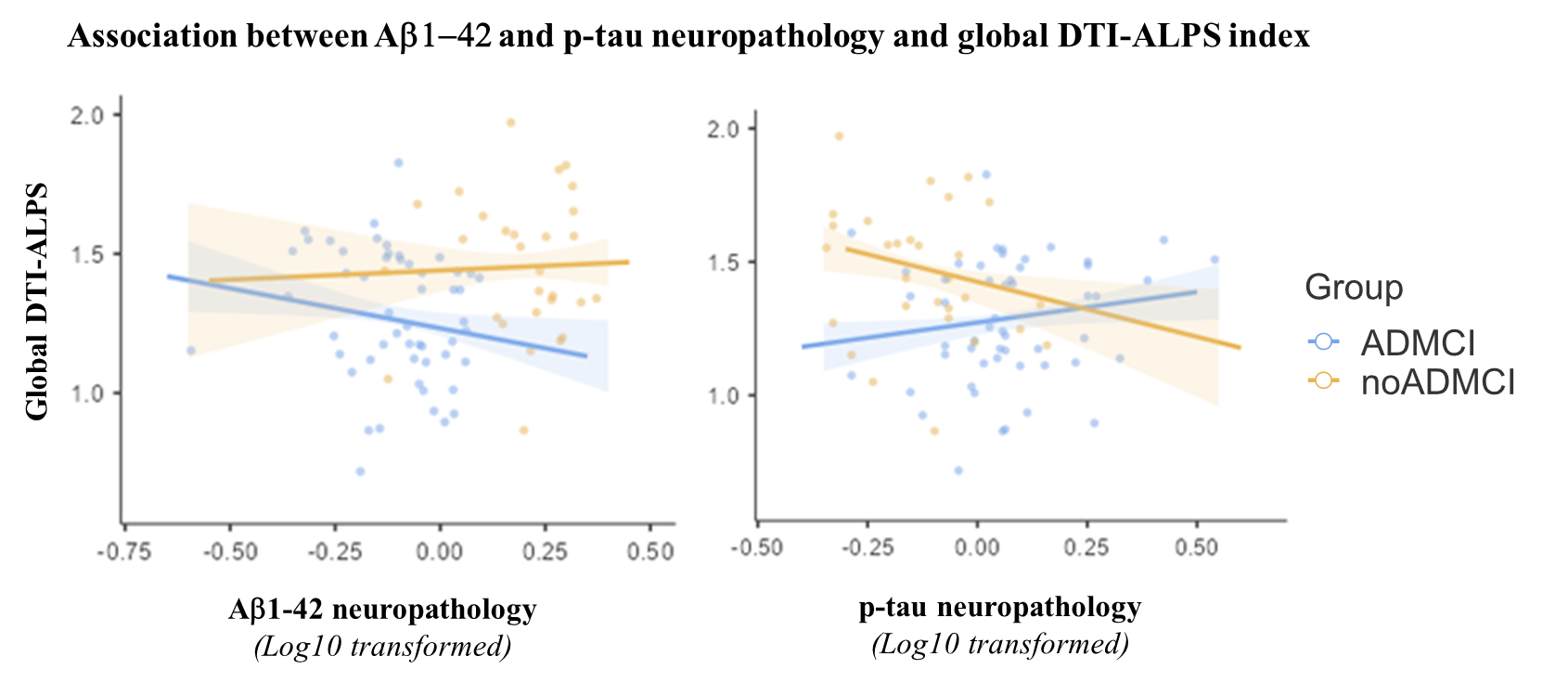
*

**Figure SM3.** *Association between Aβ1−42 and p-tau neuropathology and the global DTI-ALPS index.* The scatterplots illustrate the association between the Aβ1−42 and p-tau neuropathology and Group (as predictors) and the global DTI-ALPS index (target variable; estimated marginal means), as revealed by GLM, in the ADMCI and noADMCI participants. No statistically significant main (Aβ1-42 or p-tau) or interacting effect (Group X Aβ1-42 or p-tau) was observed including the Aβ1-42 (p = 0.607 and p = 0.410, respectively) or the p-tau (p = 0.629 and p = 0.095, respectively) and the Group factors was observed. Standard error mean (SE) is depicted as a shadowed area. *Legend: AD = Alzheimer’s disease; ADMCI = mild cognitive impairment due to AD; noADMCI = mild cognitive impairment not due to AD; DTI-ALPS = diffusion tensor imaging along the perivascular space index; Aβ1−42 = amyloid beta 1-42 markers in the CSF; p-tau = phosphorylated tau marker in the CSF; GLM = general linear regression model.*

*Periodic and aperiodic components of the rsEEG PSD in Nold, noADMCI and ADMCI participants*

Figure SM4 illustrates the grand average of the raw, aperiodic-fitted and corrected rsEEG PSD obtained from the spectral parametrization in the Nold, noADMCI and ADMCI groups. Notably, the ADMCI participants showed a prominent reduction of the corrected rsEEG PSD within the alpha frequency band (namely 8-12 Hz) compared to noADMCI participants. Both the ADMCI and noADMCI participants showed an evident reduction of the corrected rsEEG PSD within the alpha frequency band compared to the Nold group.

*
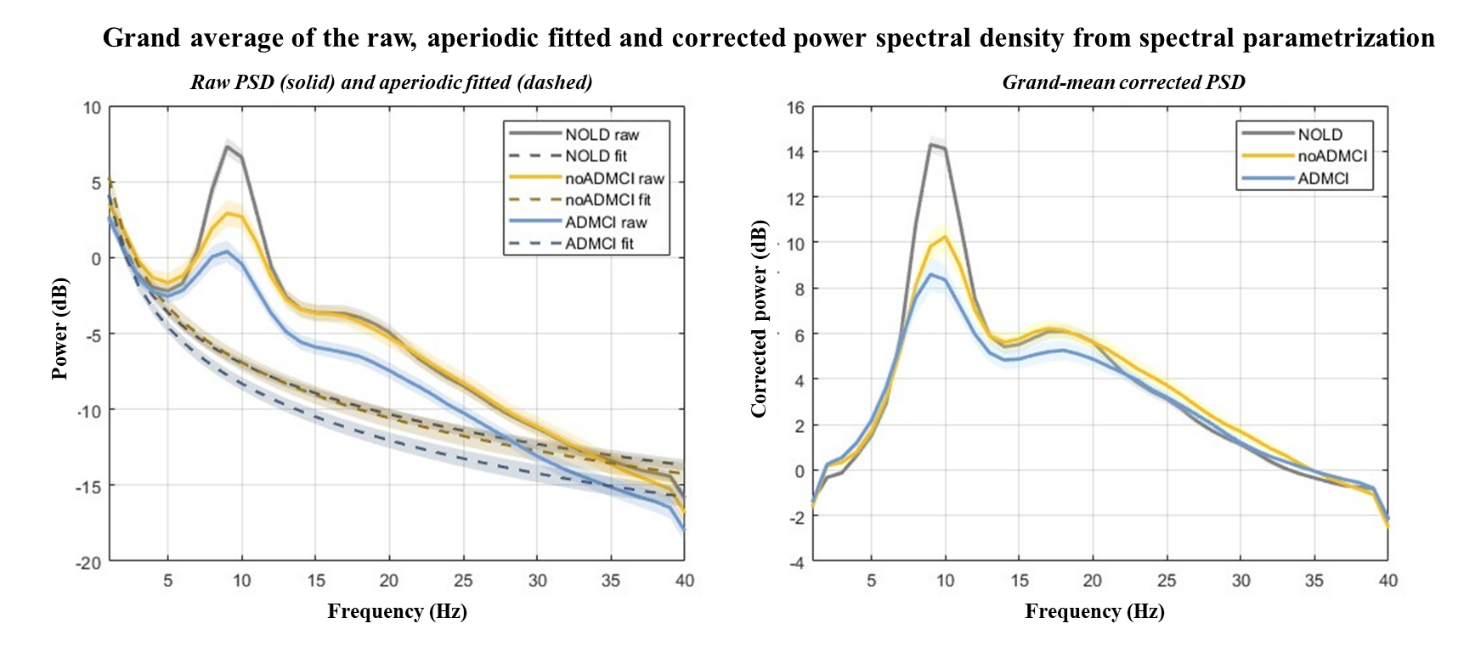
*

**Figure SM4**. *Aperiodic and periodic components of rsEEG PSD.* *Left:* Grand average of the raw (full line) and aperiodic fitted (dashed line) power spectral density (PSD; ± standard error mean, SE as shadowed area) obtained from the application of the spectral parametrization algorithm to extract the aperiodic and periodic components of the rsEEG PSD in the Nold, ADMCI and noADMCI groups. *Right*: Grand average of the corrected rsEEG PSD (± standard error mean, SE as shadowed area) in the ADMCI and noADMCI groups. *Legend: Nold = older cognitively unimpaired; AD = Alzheimer’s disease; ADMCI = mild cognitive impairment due to AD; noADMCI = mild cognitive impairment not due to AD; rsEEG = resting-state electroencephalographic; PSD = power spectral density.*

Figure SM5 illustrates the individual distribution of IAFp identified by the *specparam* procedure. Notably, the IAFp values spanned in the following ranges: for the ADMCI group = 6.1-12.6 Hz, for the noADMCI group = 6.1-12.6 Hz, and for the Nold group = 6.1-10.3 Hz.


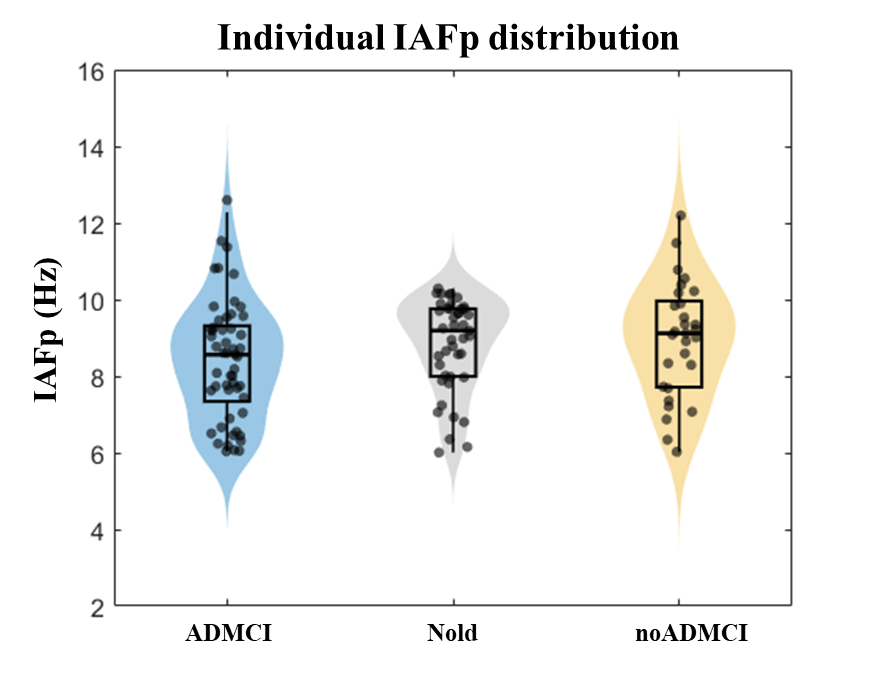


**Figure SM5**. *Individual Alpha Frequency peak identified on the aperiodic-corrected rsEEG PSD by the specparam.* Distribution of the individual values of the IAFp across groups. No outlier values were identified by the iterative (leave-one-out) Grubbs’ test to detect outliers (threshold of p > 0.001). *Legend: Nold = older cognitively unimpaired; AD = Alzheimer’s disease; ADMCI = mild cognitive impairment due to AD; noADMCI = mild cognitive impairment not due to AD; rsEEG = resting-state electroencephalographic; PSD = power spectral density.*

No statistically significant ANOVA main (Group) or interaction (Group X ROI) effects were observed for the exponent (p = 0.081 and p = 0.438, respectively) and offset variables (p = 0.201 and p = 0.155, respectively), showing no differences between the Nold, ADMCI and noADMCI groups (Figure SM6). Even excluding the Nold group, no statistically significant Group main or interaction ANOVA effects were observed for the exponent (p = 0.950 and p = 0.744, respectively) and offset variables (p = 0.116 and p = 0.505, respectively), showing no differences between ADMCI and noADMCI groups.

*
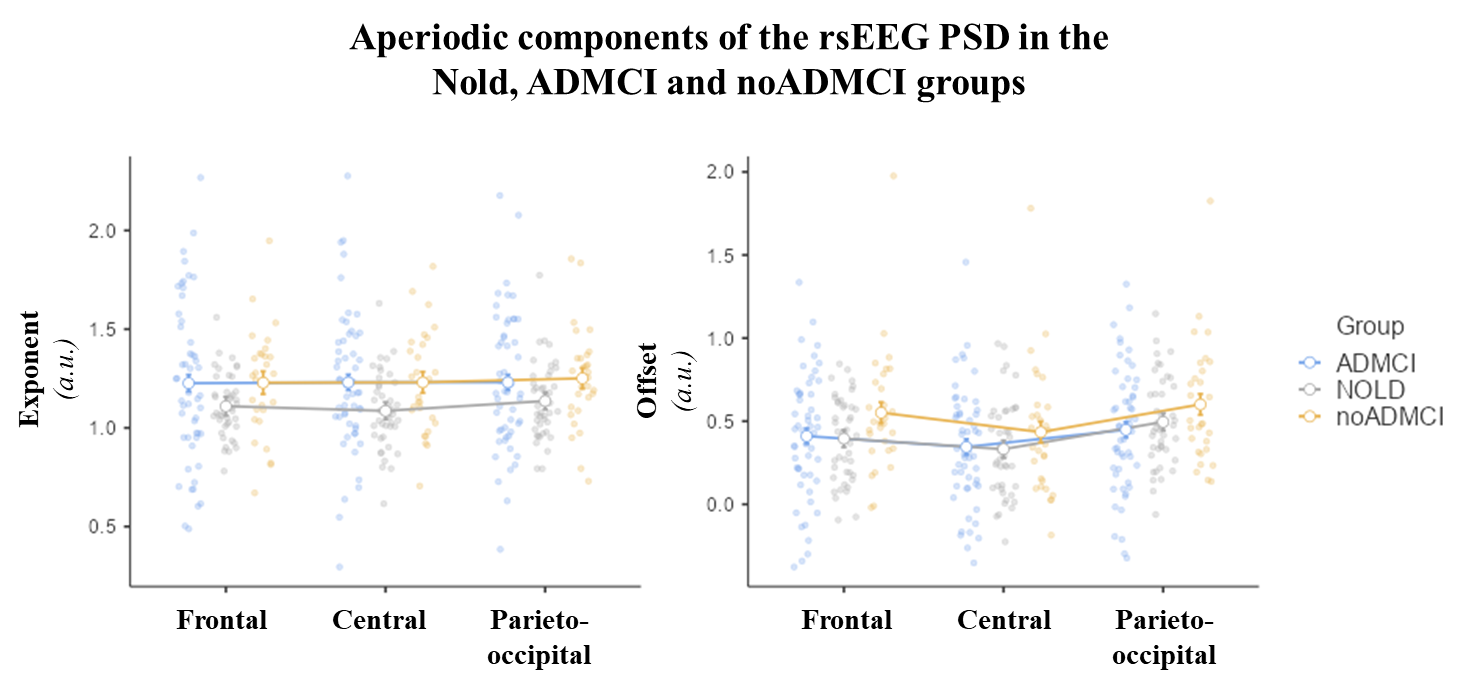
*

**Figure SM6**. *Mean values (± standard error mean, SE) of the regional aperiodic components (left: exponent, right: offset) of the rsEEG PSD.* No statistically significant ANOVA main or interaction Group X ROI effects were observed for the exponent (p = 0.081 and p = 0.438, respectively) and offset variables (p = 0.201 and p = 0.155, respectively), showing no differences between the Nold, ADMCI and noADMCI groups. *Legend: Nold = older cognitively unimpaired; AD = Alzheimer’s disease; ADMCI = mild cognitive impairment due to AD; noADMCI = mild cognitive impairment not due to AD; rsEEG = resting-state electroencephalographic; PSD = power spectral density.*

A statistically significant 3-way ANOVA interaction (F(12, 732) = 5.95, p = 0.005) among the factors Group (Nold, ADMCI, noADMCI), Band (delta, theta, low alpha, high alpha), and ROI (frontal, central, parieto-occipital). Compared to the Nold, the ADMCI and noADMCI groups were characterized by lower periodic parieto-occipital high alpha rsEEG PSD, as revealed by Duncan post-hoc testing (p < 0.05 FDR-corrected). Furthermore, compared to the Nold, the ADMCI group was characterized by lower periodic widespread low and high alpha rsEEG PSD, as revealed by Duncan post-hoc testing (p < 0.05 FDR-corrected; Figure SM7).

*
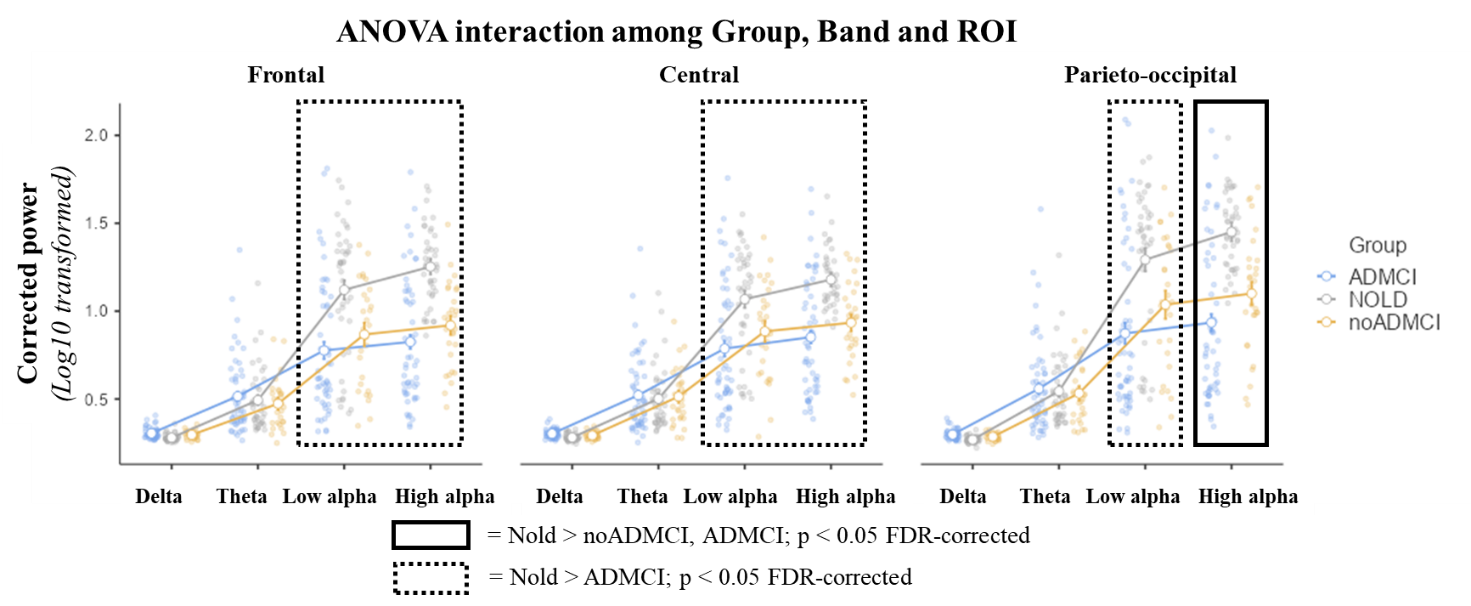
*

**Figure SM7**. *Regional periodic components of the rsEEG PSD.* Mean values (± standard error mean, SE) of periodic rsEEG PSD for (1) three groups (Nold, ADMCI, noADMCI), (2) three ROIs (frontal, central, parieto-occipital), and (3) four frequency bands (delta, theta, low alpha, high alpha). The ANOVA showed a statistically significant 3-way ANOVA interaction (F(12, 732) = 5.95, p = 0.005) among the factors Group (Nold, ADMCI, noADMCI), Band (delta, theta, low alpha, high alpha), and ROI (frontal, central, parieto-occipital). The aperiodic-corrected rsEEG PSD were used to identify the individual alpha frequency peak (IAFp) defined as the frequency showing the maximum corrected PSD value between 5 and 15 Hz from posterior electrodes (P3, Pz, P4, P7, P8, O1, O2). The frequency bands were: delta (1-3 Hz), theta (4-7 Hz), low and high alpha. These alpha sub-bands were identified as follows: the low (from IAFp-2 Hz to IAFp; 5 Hz as the lower limit) and the high (from IAFp to IAFp + 2 Hz; 13 Hz as the upper limit) alpha bands. The aperiodic-corrected rsEEG PSD were calculated in the following scalp regions of interest (ROI): frontal (Fp1, Fp2, F3, Fz, F4, F7, F8), central (T7, T8, C3, Cz, C4), and posterior (P3, Pz, P4, P7, P8, O1, O2). Compared to the Nold, the ADMCI and noADMCI groups were characterized by lower periodic parieto-occipital high alpha rsEEG PSD, as revealed by Duncan post-hoc testing (p < 0.05 FDR-corrected). Furthermore, compared to the Nold, the ADMCI group was characterized by lower periodic widespread low and high alpha rsEEG PSD, as revealed by Duncan post-hoc testing (p < 0.05 FDR-corrected). *Legend: Nold = older cognitively unimpaired; AD = Alzheimer’s disease; ADMCI = mild cognitive impairment due to AD; noADMCI = mild cognitive impairment not due to AD; rsEEG = resting-state electroencephalographic; PSD = power spectral density.*

As some participants exhibit a IAFp below 8 Hz, we compared the eyes-closed to eyes open condition of these participants to demonstrate the reactivity to eye opening (i.e., desynchronization) of low alpha rsEEG PSD, justifying the definition as low alpha sub-band (typically, “reactive” to eye opening) and not theta band (typically, not “reactive to eyes opening). Furthermore, we included a control analysis focusing on those ADMCI and noADMCI participants whose IAFp was below 7.5 Hz by comparing the eyes-open and eyes-closed conditions to calculate the reactivity to eye opening, that is a specific feature of the rsEEG alpha rhythms.

Overall, 20 participants (14 ADMCI and 6 noADMCI participants) exhibited an IAFp lower than 7.5 Hz. Of those 11 (10 ADMCI and 1 noADMCI) participants have the eyes-open condition available and were included in the analysis. Eyes-open rsEEG data underwent to the same procedure as described in the main rsEEG preprocessing and spectral analysis with spectral parametrization. Figure SM8 illustrates the individual values of the global low alpha and high alpha rsEEG aperiodic-corrected rsEEG PSD for the two conditions. A statistically significant ANOVA interaction was observed between the Band (low alpha, high alpha) and Condition (eyes-closed, eyes open) factors ( F(1, 20) = 40.41, p = 0.002), illustrating lower periodic global rsEEG PSD during eyes open as compared to eyes-closed condition for both sub-bands (low alpha: p = 0.002, high alpha: p = 0.001; FDR-corrected Duncan post-hoc testing).


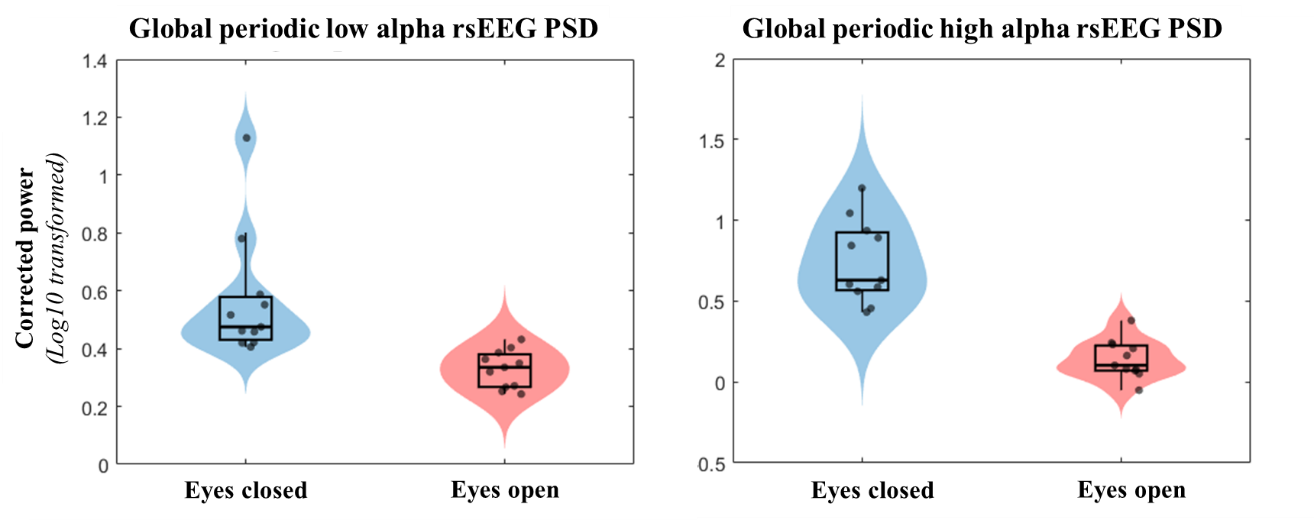


**Figure SM8**. *Individual values of the periodic global low alpha and high alpha rsEEG PSD.* The individual values of 11 participants (10 ADMCI and 1 noADMCI) exhibiting an IAFp lower than 7.5 Hz and having the eyes-open rsEEG recording available are reported. A statistically significant ANOVA interaction was observed between the Band (low alpha, high alpha) and Condition (eyes-closed, eyes open) factors ( F(1, 20) = 40.41, p = 0.002), illustrating lower periodic global rsEEG PSD during eyes open as compared to eyes-closed condition for both sub-bands (low alpha: p = 0.002, high alpha: p = 0.001; FDR-corrected Duncan post-hoc testing). No outlier values were identified by the iterative (leave-one-out) Grubbs’ test to detect outliers (threshold of p > 0.001). *Legend: AD = Alzheimer’s disease; ADMCI = mild cognitive impairment due to AD; noADMCI = mild cognitive impairment not due to AD; rsEEG = resting-state electroencephalographic; PSD = power spectral density.*

*Association between global DTI-ALPS and aperiodic rsEEG components*

Figure SM9 illustrates the association between the Group and the global DTI-ALPS index (as predictors) and the global exponent and offset as target variables (one model for each variable) in the noADMCI and ADMCI groups. No statistically significant effects (including main or interaction effect) between the DTI-ALPS index and the Group factors was observed for the exponent (standardized β = 0.123, p = 0.371) or offset (standardized β = -0.157, p = 0.252) aperiodic rsEEG parameters


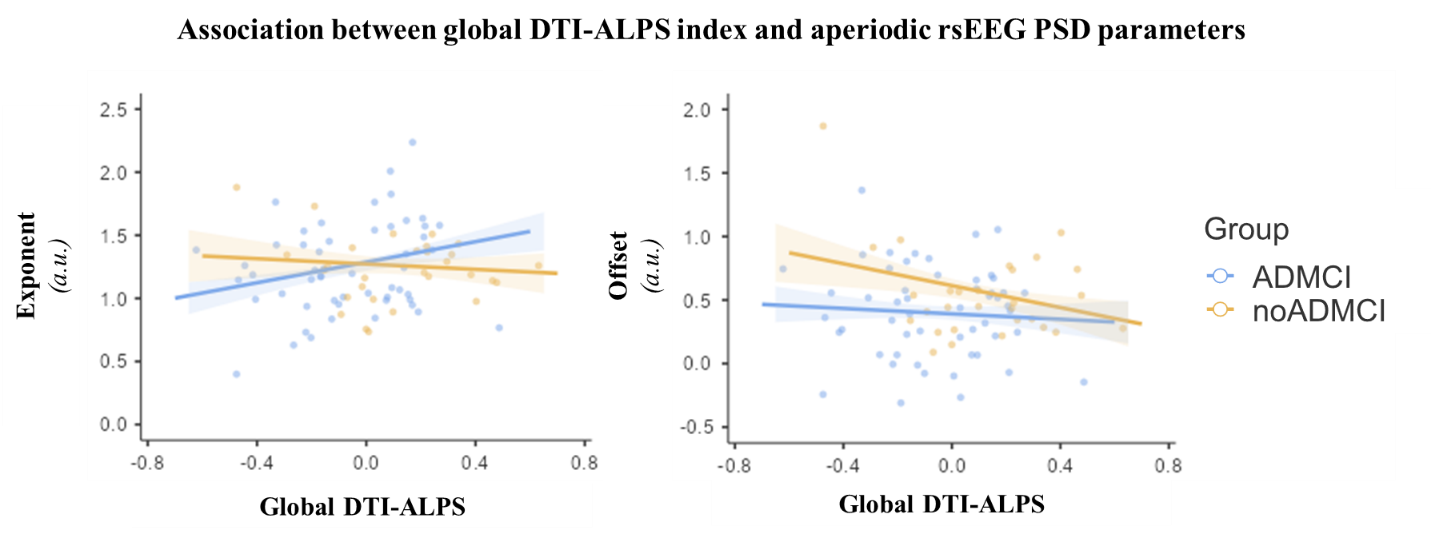


**Figure SM9**. *Association between the global DTI-ALPS index and the global aperiodic components (left: exponent, right: offset) of the rsEEG PSD.* The scatterplots illustrate the association between the global DTI-ALPS index and Group (as predictors) and the global exponent and offset (target variable; estimated marginal means), as revealed by GLM, in the ADMCI and noADMCI participants. No statistically significant interaction effect between the exponent and offset aperiodic rsEEG parameters and the Group factors was observed. Standard error mean (SE) is depicted as a shadowed area. *Legend: AD = Alzheimer’s disease; ADMCI = mild cognitive impairment due to AD; noADMCI = mild cognitive impairment not due to AD; rsEEG = resting-state electroencephalographic; PSD = power spectral density.*

*Control analysis with other spectral parametrization parameters*

Due to the current discussion in the research community about the frequency window for the most proper application of the spectral parametrization algorithm, we developed two control analyses to check the robustness of the spectral parametrization results by minimizing the potential influence of artifacts. Excluding the higher-frequency range (30–40 Hz) aimed to reduce the possible contamination from residual muscular activity, while excluding the lowest frequencies (< 3 Hz) allowed us to verify that the results were not biased by residual slow drifts or low-frequency noise components. We performed the same analysis focusing only on ADMCI and noADMCI participants as that showed in the main section.

We reported the results of two cross-validation sessions on the results of the spectral parametrization to extract the periodic (PSD peaks) and aperiodic components of the rsEEG PSD using as input:

- the absolute PSD values for frequency bins ranging from 1 to 30 Hz

- the absolute PSD values for frequency bins ranging from 3 to 30 Hz.

Figure SM10 illustrates the grand average of the raw, aperiodic-fitted and corrected rsEEG PSD obtained from the spectral parametrization in the noADMCI and ADMCI groups with the different frequency range: 1-30 Hz (upper row) and 3-30 Hz (bottom row). Notably, the ADMCI participants showed a prominent reduction of the corrected rsEEG PSD within the alpha frequency band (namely 8-12 Hz) with both frequency ranges.


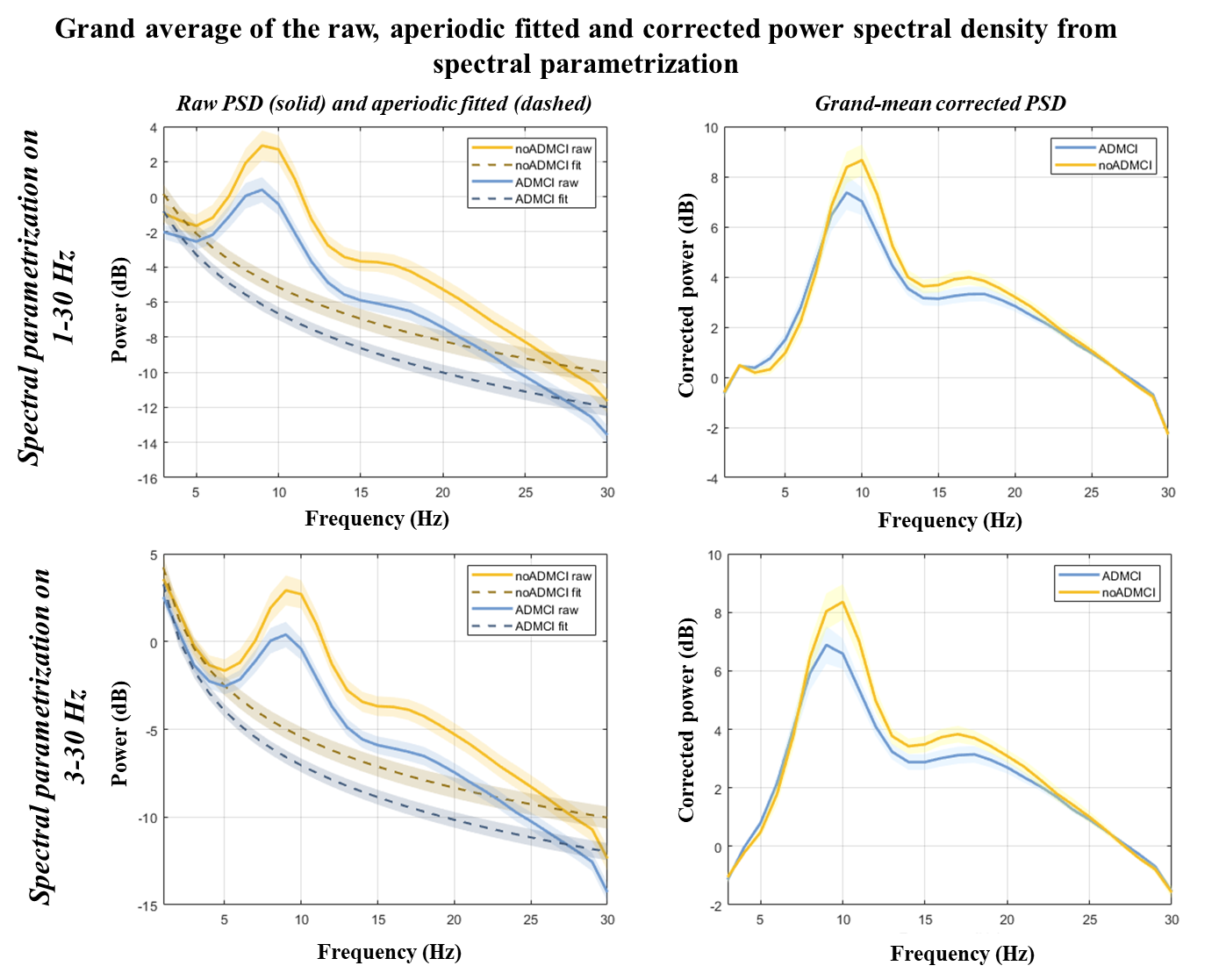


**Figure SM10**. *Aperiodic and periodic components of the rsEEG PSD with two other frequency ranges (upper row: 1-30 Hz; bottom row: 3-30 Hz)).* *Left:* Grand average of the raw (full line) and aperiodic fitted (dashed line) power spectral density (PSD; ± standard error mean, SE as shadowed area) obtained from the application of the spectral parametrization algorithm to obtain the aperiodic and periodic components of the rsEEG PSD in the ADMCI and noADMCI groups. *Right*: Grand average of the corrected rsEEG PSD (± standard error mean, SE as shadowed area) in the ADMCI and noADMCI groups. *Legend: AD = Alzheimer’s disease; ADMCI = mild cognitive impairment due to AD; noADMCI = mild cognitive impairment not due to AD; rsEEG = resting-state electroencephalographic; PSD = power spectral density.*

The results confirmed the main findings (data not shown), namely:

- no differences concerning the regional aperiodic components (exponent and offset) of the rsEEG PSD between the ADMCI and noADMCI groups;

- the ADMCI group was characterized by abnormally lower posterior periodic alpha rsEEG PSD as compared to the noADMCI group;

- the global and posterior periodic alpha rsEEG PSD was associated with DTI-ALPS index (no Group effect).

*Control analysis with other DTI-ALPS estimation methods*

Given the ongoing debate about the specificity and interpretability of the DTI-ALPS index^1^, we performed a complementary analysis using an alternative spatial normalization strategy for DTI-ALPS estimation. The same DTI preprocessing pipeline described above was applied, with the only difference that spatial normalization was performed directly on the diffusion tensor–derived maps without using individual T1-weighted images. In this approach, individual FA maps were registered to JHU-ICBM-FA-1mm template in standard space using FSL registration tools. The resulting affine (FSL *flirt*) and nonlinear (FSL *fnirt*) transformations were then applied to the diffusion tensor–derived maps using FSL *applywarp*, bringing Dxx, Dyy, and Dzz into JHU-ICBM-FA template space for subsequent ALPS calculations.

The results confirmed the main findings, namely:

- Higher global DTI-ALPS index in the noADMCI group as compared to the ADMCI group, as revealed by a main Group effect (F(1, 80) = 10.8; p = 0.002; Figure SM11). ADMCI group was characterized by lower left and right DTI-ALPS indexes (p = 0.013 and p = 0.018, respectively, Duncan post-hoc FDR-corrected) as compared to the noADMCI group.


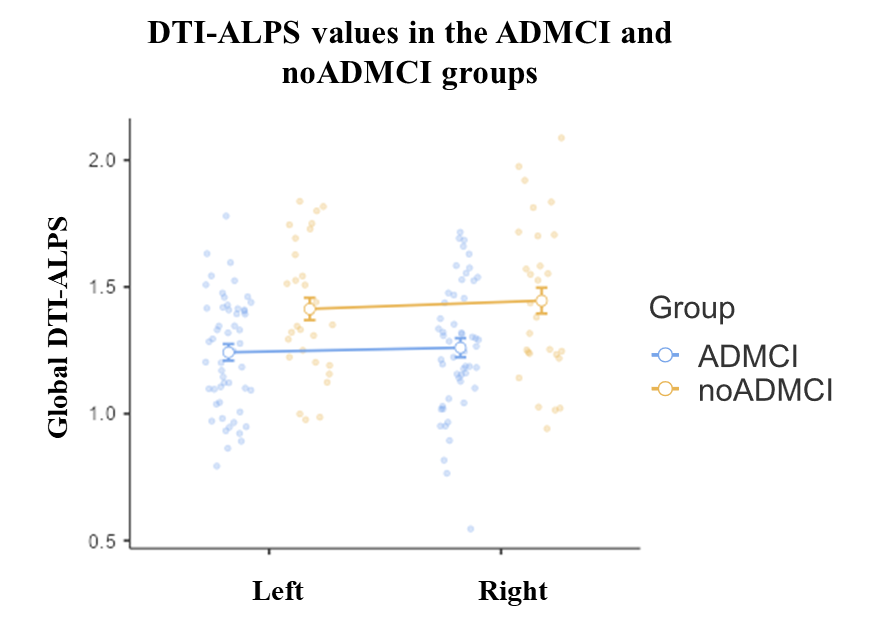


**Figure SM11.** *Mean values (± standard error mean, SE) of the left, right and global (average between left and right) DTI-ALPS index (calculated according to cross-validation method).* No statistically significant 2-way ANOVA interaction between the Group (ADMCI, noADMCI), and ROI (Left, Right) factors was observed (p = 0.761). A main Group effect (F(1,80) = 10.8; p = 0.002) was observed, showing that ADMCI group was characterized by lower left and right DTI-ALPS indexes (p = 0.013 and p = 0.018, respectively, Duncan post-hoc FDR-corrected) as compared to the noADMCI group. *Legend: AD = Alzheimer’s disease; ADMCI = mild cognitive impairment due to AD; noADMCI = mild cognitive impairment not due to AD; DTI-ALPS = diffusion tensor imaging along the perivascular space.*

- Higher global DTI-ALPS index associated with fewer white matter lesions (Figure SM12), higher global and posterior periodic alpha rsEEG PSD (Figure SM13), and better cognitive performance (Figure SM14), in both groups (no Group effects);


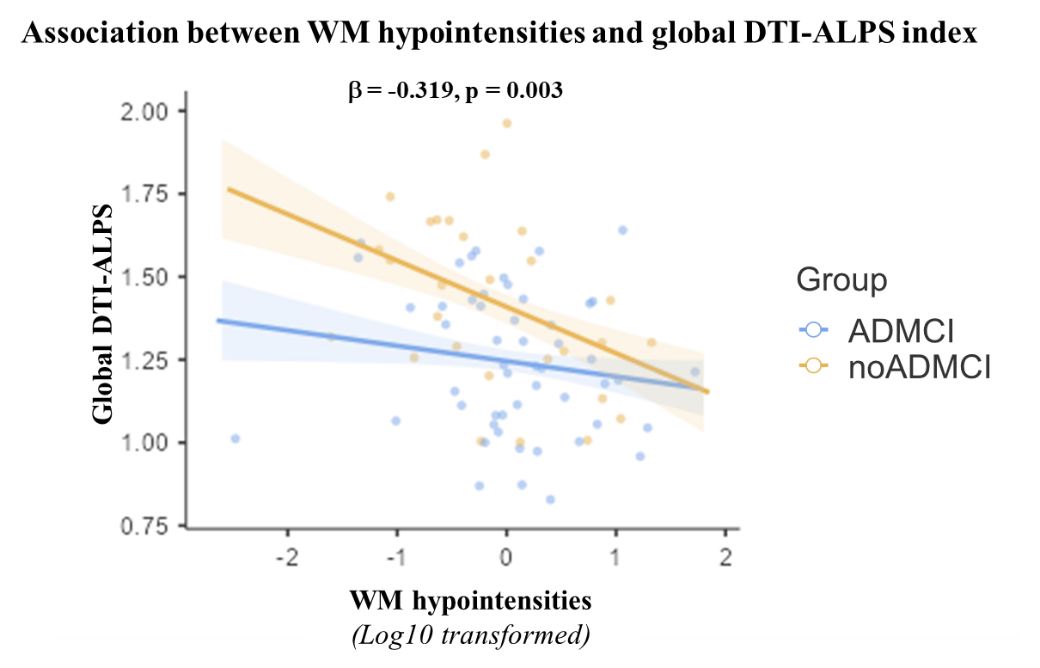


**Figure SM12.** *Association between white matter (WM) hypointensities and the global DTI-ALPS index (calculated according to cross-validation method).* The scatterplots illustrate the association between the WM hypointensities and Group (as predictors) and the global DTI-ALPS index (target variable; estimated marginal means), as revealed by GLM, in the ADMCI and noADMCI participants. Only the main effect of the WM hypointensities was statistically significant (standardized β = -0.319, p = 0.003). Standard error mean (SE) is depicted as a shadowed area. *Legend: AD = Alzheimer’s disease; ADMCI = mild cognitive impairment due to AD; noADMCI = mild cognitive impairment not due to AD; DTI-ALPS = diffusion tensor imaging along the perivascular space index; WM = white matter; GLM = general linear regression model.*


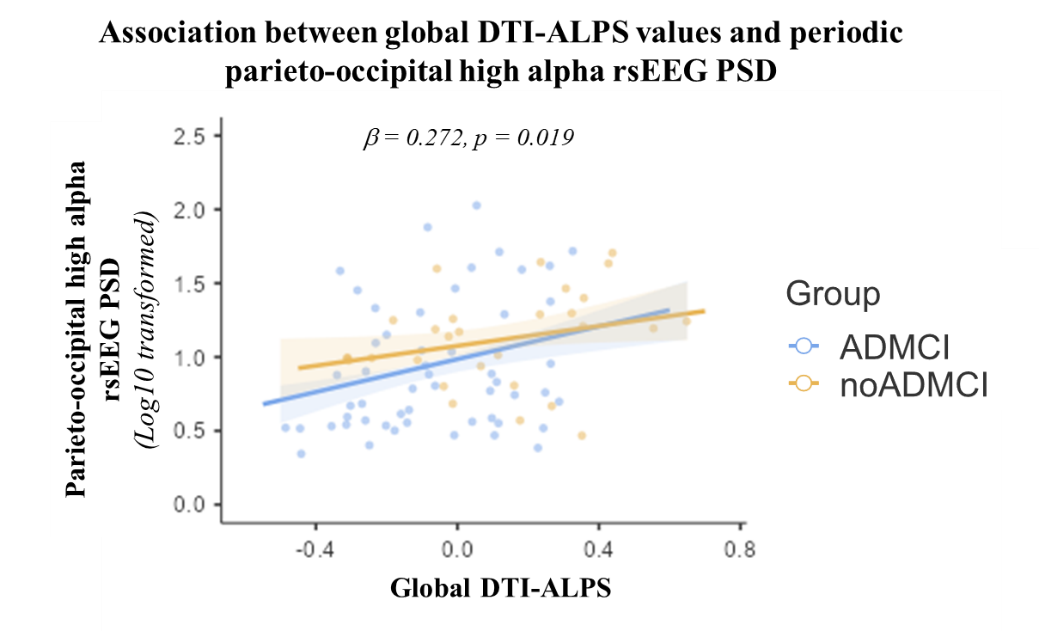


**Figure SM13.** *Association between global DTI-ALPS (calculated according to cross-validation method) and periodic rsEEG PSD*. The scatterplots illustrate the association between the global DTI-ALPS index and Group (as predictors) and the periodic parieto-occipital high alpha rsEEG PSD (target variable; estimated marginal means), as revealed by GLM, in the ADMCI and noADMCI participants. Only the main effect of the global DTI-ALPS index was statistically significant parieto-occipital high alpha (standardized β = 0.272, p = 0.019). Standard error mean (SE) is depicted as a shadowed area. *Legend: AD = Alzheimer’s disease; ADMCI = mild cognitive impairment due to AD; noADMCI = mild cognitive impairment not due to AD; DTI-ALPS = diffusion tensor imaging along the perivascular space; rsEEG = resting-state electroencephalographic; PSD = power spectral density.*


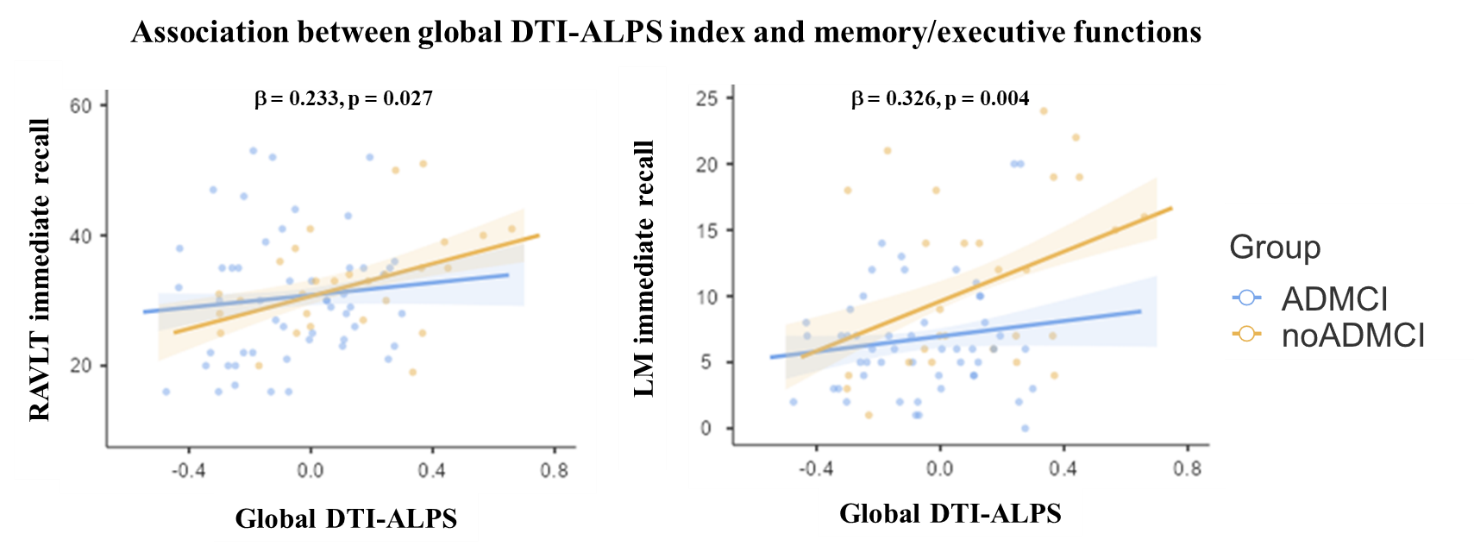


**Figure SM14.** *Association between global DTI-ALPS (calculated according to cross-validation method) and memory/executive function.* The scatterplots illustrate the association between the global DTI-ALPS index and Group (as predictors) and the memory/executive function (target variable; estimated marginal means), as revealed by GLM, in the ADMCI and noADMCI participants. Only the main effect of the global DTI-ALPS index was statistically significant on the Rey Auditory Verbal Learning immediate recall (standardized β = 0.233, p = 0.027) and on the Logic Memory immediate recall (standardized β = 0.326, p = 0.004). Standard error mean (SE) is depicted as shadowed area. *Legend: AD = Alzheimer’s disease; ADMCI = mild cognitive impairment due to AD; noADMCI = mild cognitive impairment not due to AD; DTI-ALPS = diffusion tensor imaging along the perivascular space; RAVLT = Rey Auditory Verbal Learning Task; LM = logic memory; GLM = general linear regression model.*

- concerning the association between the AD neuropathology and global DTI-ALPS, different trends were observed for the ADMCI (negative) and noADMCI (positive) groups (Figure SM15). This suggests that clinically relevant AD neuropathology may interfere with glymphatic function, even inverting the physiological clearance of toxic solutes, such as phosphorylated tau, through the CSF-ISF flow.


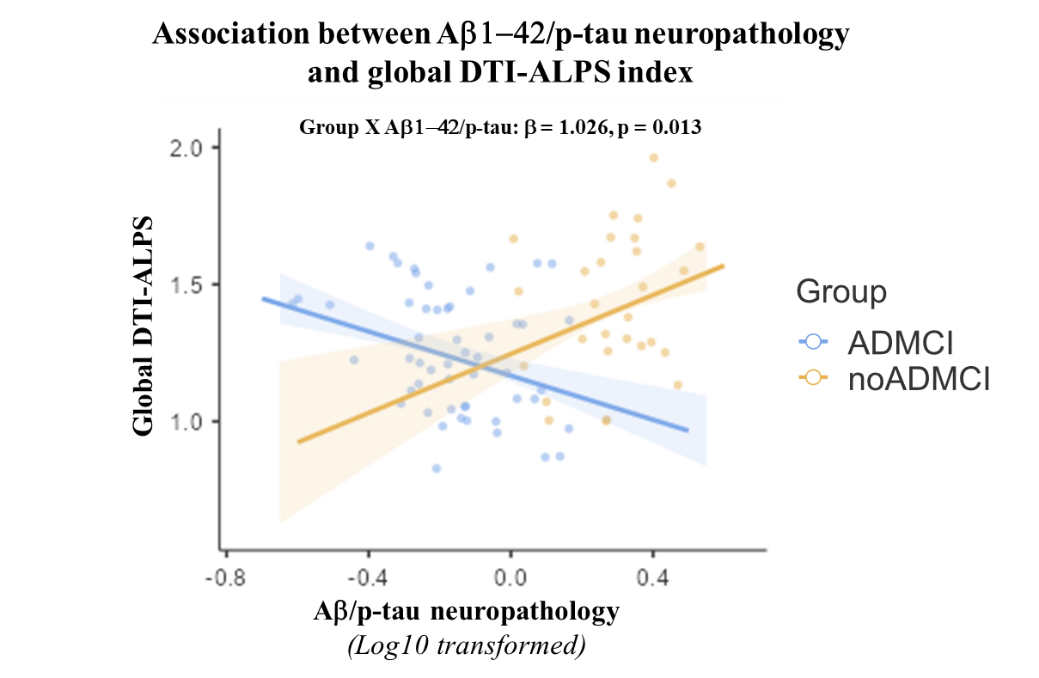


**Figure SM15.** *Association between Aβ1−42/p-tau neuropathology and the global DTI-ALPS index (calculated according to cross-validation method).* The scatterplots illustrate the association between the Aβ1−42/p-tau neuropathology and Group (as predictors) and the global DTI-ALPS index (target variable; estimated marginal means), as revealed by GLM, in the ADMCI and noADMCI participants. A statistically significant interaction effect between the Aβ/p-tau neuropathology and the Group factors was observed (standardized β = 1.026, p = 0.013). Simple effect revealed a slight statistically significant effect only for the ADMCI (standardized β = -0.408, p = 0.038), while no effect was observed for the noADMCI (standardized β = 0.584, p = 0.088). Standard error mean (SE) is depicted as shadowed area. *Legend: AD = Alzheimer’s disease; ADMCI = mild cognitive impairment due to AD; noADMCI = mild cognitive impairment not due to AD; DTI-ALPS = diffusion tensor imaging along the perivascular space index; Aβ1−42/p-tau = ratio between the amyloid beta 1-42 and phosphorylated tau markers in the CSF; GLM = general linear regression model.*

***Supplementary Material Discussion***

*Methodological remarks*

In interpreting the present results, some methodological limitations of the study design and DTI-ALPS procedures should be considered^20-22^. DTI-ALPS index is significantly influenced by white matter microstructure, including fiber crossings, undulations, and dispersion. These findings suggest that DTI-ALPS derived metrics may not provide a direct measure of glymphatic function but rather reflect underlying axonal geometry. Interpretations of DTI-ALPS-derived metrics as biomarkers of glymphatic function must consider these anatomical complexities, and future studies should integrate advanced modeling approaches to disentangle perivascular contributions from white matter structure.

(i) The technique's sensitivity and specificity are points of concern, as DTI measures bulk water movement and may not accurately reflect the dynamic interstitial fluid movements critical to glymphatic function^22^. Taoka et al.^15^ themselves have recently emphasized that the DTI-ALPS index directly indicates “*predominant Brownian motion of water molecules in the radial direction at the lateral ventricular body level, no more and no less*,” and urge careful distinction between a change in the index and an assertion of “glymphatic dysfunction.” Echoing this need for careful interpretation, and despite its growing application, the specificity of the DTI-ALPS-index for perivascular diffusion remains unclear, as various microstructural and anatomical factors may contribute to the measured signal.

(ii) Studies in humans using [intrathecal](https://www.sciencedirect.com/topics/neuroscience/intrathecal) contrast agents have demonstrated minimal CSF-ISF exchange in the deep brain WM where the DTI-ALPS index is measured. The DTI-ALPS index is significantly influenced by WM microstructure, including fiber crossings, undulations, and dispersion^20-22^. These findings suggest that other mechanisms, such as blood-brain barrier transport or proteolytic degradation, likely dominate in these areas.

(iii) The sparse presence of perivascular spaces in white matter, comprising only about 1% of tissue volume, poses another challenge. The DTI-ALPS index may struggle to differentiate water diffusivity in PVS from other sources, such as diffusion along fiber tracts.

(iv) The spatial resolution of DTI is relatively low, potentially complicating the accurate assessment of [fine structures](https://www.sciencedirect.com/topics/medicine-and-dentistry/fine-structure) within the PVS where glymphatic exchange occurs. Additionally, in DTI, the tensor model is designed to resolve a single fiber direction, which is generally effective only in brain regions like the [corpus callosum](https://www.sciencedirect.com/topics/neuroscience/corpus-callosum), where fibers largely run in parallel. However, DTI-ALPS is principally used to measure areas with intersecting fibers, a scenario where traditional DTI can be less effective.

(v) Radial asymmetry is widespread across white matter and persists even at high b-values, suggesting a dominant contribution from axonal geometry rather than faster PVS-specific diffusion. Crossing fibers significantly inflate DTI-ALPS indices, with greater radial asymmetry observed in regions with a greater prevalence of crossing fibers. Furthermore, anisotropic axonal dispersion and undulations introduce systematic asymmetry independent of perivascular diffusion. Finally, high-resolution vascular imaging reveals substantial heterogeneity in medullary vein orientation, challenging the assumption that PVS consistently aligns with the left-right axis in DTI-ALPS index regions of interest.

(vi) The sensitivity and specificity of the technique are a cause of concern, as DTI measures bulk water movement around neural fibers and may not accurately reflect the dynamic ISF movements that are critical to physiological glymphatic function^20-22^. Despite its growing application, the specificity of the DTI-ALPS index for perivascular diffusion remains unclear, as various and complex microstructural and anatomical factors may contribute to the measured signal, necessitating careful interpretation.

(vii) When the PVS structure is locally damaged (e.g., due to local edema and microstructural changes), the global glymphatic clearance, as measured by the DTI-ALPS index, may not accurately reflect local impairment of the CSF-ISF exchange. Furthermore, the brain glymphatic drainage system does not work in isolation to remove soluble waste products from the brain. Rather, various overlapping clearance systems work together to achieve and maintain parenchymal homeostasis in the brain^20-22^. Soluble waste products in the brain can be enzymatically degraded, cleared directly into the blood via the blood–brain barrier, or transported into the CSF by a variety of mechanisms, including glymphatic clearance. Given the complexity of these mechanisms, future studies should be designed to provide a conclusive explanation for the present results.

Other limitations are related to the rsEEG acquisition system:

(i) In this exploratory study, clinical and rsEEG datasets were obtained from several clinical units, not all of which underwent a preliminary rigorous phase of standardizing operating procedures for data collection in a prospective clinical trial. The same preprocessing and spectral parameterization pipeline was uniformly applied across all EEG datasets from the participating centers, with no specific harmonization procedure such as *ComBat* or advanced harmonization statistics.

(ii) The current study employed the 10–20 montage system, utilizing 19 scalp electrodes for rsEEG recordings. This electrode configuration is considered appropriate for exploratory retrospective rsEEG studies involving ADMCI/noADMCI patients, especially because we analyzed only scalp EEG signals, without estimation of source activities. Due to spatial resolution limitation, the aperiodic and periodic components of the rsEEG power spectral density (PSD) were estimated within broad cortical regions of interest instead of a fine single electrode localization. Future studies should use high-resolution EEG techniques with 64–256 scalp electrodes to reach a high spatial resolution in the rsEEG source estimation.

(iii) The study results showed that AD-related neuropathology significantly affects such activity in ADMCI patients at the low spatial resolution allowed by the general methodology and encourages the modeling of the underlying functional cortical connectivity in future studies using EEG recordings with a higher number of scalp electrodes (>30 electrodes).

(iv) Regarding the rsEEG data analysis, the 19-channel montage may not have provided sufficient spatial resolution to capture localized topographical patterns fully. Furthermore, several methodological considerations pertain to the spectral parameterization approach rather than alternatives that offer specific features. In the present study, we used the *specparam* platform (“FOOOF”) platform (<https://specparam-tools.github.io/>)^17^ rather than alternatives that offer specific features. Specifically, the algorithm known as PaWNextra explicitly separates pink (1/f) and white noise components from the observed EEG power spectra and subtracts them from the recorded EEG activity for post-processing analysis of oscillatory components^23,24^. Another procedure, the nonparametric irregular resampling auto-spectral analysis (IRASA), separates fractal and oscillatory components in the EEG activity^25^. These approaches may address a methodological weakness of *specparam*, which can fit aperiodic components to EEG power spectra that locally exceed the empirical EEG power, producing negative residual values in the flattened (aperiodic-corrected) EEG power spectra. These residuals are expressed in arbitrary units and reflect model discrepancies rather than true negative power values. Moreover, *specparam* does not explicitly distinguish white from pink noise contributions to the EEG power spectra, so its solutions may be relatively sensitive to high-frequency noise. For this aspect, the present 1–40 Hz fit range and the study’s focus on resting-state may mitigate the influence of high-frequency spectral EEG components.

(v) In addition, the experimental design did not include external stimuli and cognitive–motor demands to provide behavioral measures of vigilance to be correlated with the EEG data. At this early research stage, these demands were not included to avoid interferences with the scope of the resting-state condition (e.g., induce spontaneous EEG activity).

Finally, this study was cross-sectional and examined periodic and aperiodic rsEEG power density spectra in a multicenter cohort of ADMCI, noADMCI, and Nold participants. The number of ADMCI and noADMCI participants was relatively small, limiting subgroup analyses by cardiovascular or metabolic risk factors, socioeconomic factors, or lifestyle factors (e.g., daily physical exercise) and hiding some relevant results on aperiodic components for the lack of statistical power. Overall, future field studies should rely on larger, heterogeneous, longitudinal ADMCI and noADMCI cohorts, with assessments of CSF, rsEEG rhythms, and MRI data to analyze DTI-ALPS and WML, with a particular focus on the neuroinflammation and neurodegeneration in the neuromodulatory subcortical systems implicated in vigilance regulation.

***References***

1. Del Percio, C.; Lizio, R.; Lopez, S.; Noce, G.; Carpi, M.; Jakhar, D.; Soricelli, A.; Salvatore, M.; Yener, G.; Güntekin, B.; Massa, F.; Arnaldi, D.; Famà, F.; Pardini, M.; Ferri, R.; Carducci, F.; Lanuzza, B.; Stocchi, F.; Vacca, L.; Coletti, C.; Marizzoni, M.; Taylor, J. P.; Hanoğlu, L.; Yılmaz, N. H.; Kıyı, İ.; Özbek-İşbitiren, Y.; D’Anselmo, A.; Bonanni, L.; Biundo, R.; D’Antonio, F.; Bruno, G.; Antonini, A.; Giubilei, F.; Farotti, L.; Parnetti, L.; Frisoni, G. B.; Babiloni, C. Resting-State EEG Alpha Rhythms Are Related to CSF Tau Biomarkers in Prodromal Alzheimer’s Disease. *Int J Mol Sci* **2025**, *26* (1), 356. https://doi.org/10.3390/ijms26010356.
2. Jack, C. R.; Andrews, J. S.; Beach, T. G.; Buracchio, T.; Dunn, B.; Graf, A.; Hansson, O.; Ho, C.; Jagust, W.; McDade, E.; Molinuevo, J. L.; Okonkwo, O. C.; Pani, L.; Rafii, M. S.; Scheltens, P.; Siemers, E.; Snyder, H. M.; Sperling, R.; Teunissen, C. E.; Carrillo, M. C. Revised Criteria for Diagnosis and Staging of Alzheimer’s Disease: Alzheimer’s Association Workgroup. *Alzheimer’s & Dementia* **2024**, *20* (8), 5143–5169. https://doi.org/10.1002/alz.13859.
3. Nathan, P. J.; Lim, Y. Y.; Abbott, R.; Galluzzi, S.; Marizzoni, M.; Babiloni, C.; Albani, D.; Bartres-Faz, D.; Didic, M.; Farotti, L.; Parnetti, L.; Salvadori, N.; Müller, B. W.; Forloni, G.; Girtler, N.; Hensch, T.; Jovicich, J.; Leeuwis, A.; Marra, C.; Molinuevo, J. L.; Nobili, F.; Pariente, J.; Payoux, P.; Ranjeva, J.-P.; Rolandi, E.; Rossini, P. M.; Schönknecht, P.; Soricelli, A.; Tsolaki, M.; Visser, P. J.; Wiltfang, J.; Richardson, J. C.; Bordet, R.; Blin, O.; Frisoni, G. B. Association between CSF Biomarkers, Hippocampal Volume and Cognitive Function in Patients with Amnestic Mild Cognitive Impairment (MCI). *Neurobiology of Aging* **2017**, *53*, 1–10. https://doi.org/10.1016/j.neurobiolaging.2017.01.013.
4. Fischl, B. Automatically Parcellating the Human Cerebral Cortex. *Cerebral Cortex* **2004**, *14* (1), 11–22. https://doi.org/10.1093/cercor/bhg087.
5. Reuter, M.; Schmansky, N. J.; Rosas, H. D.; Fischl, B. Within-Subject Template Estimation for Unbiased Longitudinal Image Analysis. *NeuroImage* **2012**, *61* (4), 1402–1418. https://doi.org/10.1016/j.neuroimage.2012.02.084.
6. Wei, N.; Deng, Y.; Yao, L.; Jia, W.; Wang, J.; Shi, Q.; Chen, H.; Pan, Y.; Yan, H.; Zhang, Y.; Wang, Y. A Neuroimaging Marker Based on Diffusion Tensor Imaging and Cognitive Impairment Due to Cerebral White Matter Lesions. *Front. Neurol.* **2019**, *10*, 81. https://doi.org/10.3389/fneur.2019.00081
7. Tournier, J.-D.; Smith, R.; Raffelt, D.; Tabbara, R.; Dhollander, T.; Pietsch, M.; Christiaens, D.; Jeurissen, B.; Yeh, C.-H.; Connelly, A. MRtrix3: A Fast, Flexible and Open Software Framework for Medical Image Processing and Visualisation. *NeuroImage* **2019**, *202*, 116137. https://doi.org/10.1016/j.neuroimage.2019.116137.
8. Jenkinson M, Beckmann CF, Behrens TE, Woolrich MW, Smith SM. FSL. *Neuroimage*. **2012**;62(2):782-790. doi:10.1016/j.neuroimage.2011.09.015
9. Li X, Morgan PS, Ashburner J, Smith J, Rorden C. The first step for neuroimaging data analysis: DICOM to NIfTI conversion. *J Neurosci Methods.* **2016**;264:47-56. doi:10.1016/j.jneumeth.2016.03.001
10. Veraart J, Novikov DS, Christiaens D, Ades-Aron B, Sijbers J, Fieremans E. Denoising of diffusion MRI using random matrix theory. *Neuroimage*. **2016**;142:394-406. doi:10.1016/j.neuroimage.2016.08.016
11. Kellner E, Dhital B, Kiselev VG, Reisert M. Gibbs-ringing artifact removal based on local subvoxel-shifts. *Magn Reson Med*. **2016**;76(5):1574-1581. doi:10.1002/mrm.26054
12. Andersson JLR, Sotiropoulos SN. An integrated approach to correction for off-resonance effects and subject movement in diffusion MR imaging. *Neuroimage*. 2016;125:1063-1078. doi:10.1016/j.neuroimage.2015.10.019
13. Liu, X.; Barisano, G.; Shao, X.; Jann, K.; Ringman, J. M.; Lu, H.; Arfanakis, K.; Caprihan, A.; DeCarli, C.; Gold, B. T.; Maillard, P.; Satizabal, C. L.; Fadaee, E.; Habes, M.; Stables, L.; Singh, H.; Fischl, B.; Kouwe, A. V. D.; Schwab, K.; Helmer, K. G.; Greenberg, S. M.; Wang, D. J. J. Cross-Vendor Test-Retest Validation of Diffusion Tensor Image Analysis along the Perivascular Space (DTI-ALPS) for Evaluating Glymphatic System Function. *Aging and disease* **2023**, 0. https://doi.org/10.14336/AD.2023.0321-2.
14. Nepozitek, J.; Marecek, S.; Rottova, V.; Dostalova, S.; Krajca, T.; Keller, J.; Sonka, K.; Dusek, P. Glymphatic Dysfunction Evidenced by DTI-ALPS Is Related to Obstructive Sleep Apnea Intensity in Newly Diagnosed Parkinson’s Disease. *npj Parkinsons Dis.* **2025**, *11* (1), 160. https://doi.org/10.1038/s41531-025-01018-8.
15. Taoka, T.; Masutani, Y.; Kawai, H.; Nakane, T.; Matsuoka, K.; Yasuno, F.; Kishimoto, T.; Naganawa, S. Evaluation of Glymphatic System Activity with the Diffusion MR Technique: Diffusion Tensor Image Analysis along the Perivascular Space (DTI-ALPS) in Alzheimer’s Disease Cases. *Jpn J Radiol* **2017**, *35* (4), 172–178. https://doi.org/10.1007/s11604-017-0617-z.
16. Kopčanová, M.; Tait, L.; Donoghue, T.; Stothart, G.; Smith, L.; Flores-Sandoval, A. A.; Davila-Perez, P.; Buss, S.; Shafi, M. M.; Pascual-Leone, A.; Fried, P. J.; Benwell, C. S. Y. Resting-State EEG Signatures of Alzheimer’s Disease Are Driven by Periodic but Not Aperiodic Changes. *Neurobiology of Disease* **2024**, *190*, 106380. https://doi.org/10.1016/j.nbd.2023.106380.
17. Donoghue, T.; Haller, M.; Peterson, E. J.; Varma, P.; Sebastian, P.; Gao, R.; Noto, T.; Lara, A. H.; Wallis, J. D.; Knight, R. T.; Shestyuk, A.; Voytek, B. Parameterizing Neural Power Spectra into Periodic and Aperiodic Components. *Nat Neurosci* **2020**, *23* (12), 1655–1665. https://doi.org/10.1038/s41593-020-00744-x.
18. Klimesch, W. EEG Alpha and Theta Oscillations Reflect Cognitive and Memory Performance: A Review and Analysis. *Brain Res Brain Res Rev* **1999**, *29* (2–3), 169–195. https://doi.org/10.1016/s0165-0173(98)00056-3.
19. Babiloni, C.; Lopez, S.; Noce, G.; Ferri, R.; Panerai, S.; Catania, V.; Soricelli, A.; Salvatore, M.; Nobili, F.; Arnaldi, D.; Famà, F.; Massa, F.; Buttinelli, C.; Giubilei, F.; Stocchi, F.; Vacca, L.; Marizzoni, M.; D’Antonio, F.; Bruno, G.; De Lena, C.; Güntekin, B.; Yıldırım, E.; Hanoğlu, L.; Yener, G.; Yerlikaya, D.; Taylor, J. P.; Schumacher, J.; McKeith, I.; Bonanni, L.; Pantano, P.; Piervincenzi, C.; Petsas, N.; Frisoni, G. B.; Del Percio, C.; Carducci, F. Relationship between Default Mode Network and Resting-State Electroencephalographic Alpha Rhythms in Cognitively Unimpaired Seniors and Patients with Dementia Due to Alzheimer’s Disease. *Cerebral Cortex* **2023**, *33* (20), 10514–10527. https://doi.org/10.1093/cercor/bhad300.
20. Ringstad, G. Glymphatic Imaging: A Critical Look at the DTI-ALPS Index. *Neuroradiology* **2024**, *66* (2), 157–160. https://doi.org/10.1007/s00234-023-03270-2.
21. Botta, D.; Hutuca, I.; Ghoul, E. E.; Sveikata, L.; Assal, F.; Lövblad, K.-O.; Kurz, F. T. Emerging Non-Invasive MRI Techniques for Glymphatic System Assessment in Neurodegenerative Disease. *J Neuroradiol* **2025**, *52* (3), 101322. https://doi.org/10.1016/j.neurad.2025.101322.
22. Tarasoff-Conway, J. M.; Carare, R. O.; Osorio, R. S.; Glodzik, L.; Butler, T.; Fieremans, E.; Axel, L.; Rusinek, H.; Nicholson, C.; Zlokovic, B. V.; Frangione, B.; Blennow, K.; Ménard, J.; Zetterberg, H.; Wisniewski, T.; de Leon, M. J. Clearance Systems in the Brain-Implications for Alzheimer Disease. *Nat Rev Neurol* **2015**, *11* (8), 457–470. https://doi.org/10.1038/nrneurol.2015.119.
23. Barry, R. J.; De Blasio, F. M. Characterizing Pink and White Noise in the Human Electroencephalogram. *J. Neural Eng.* **2021**, *18* (3), 034001. https://doi.org/10.1088/1741-2552/abe399.
24. Jaramillo-Jimenez, A.; Mantilla-Ramos, Y.-J.; Tovar-Rios, D. A.; Lopera, F.; Aguillón, D.; Ochoa-Gomez, J. F.; Paquet, C.; Gaubert, S.; Pardini, M.; Arnaldi, D.; Taylor, J.-P.; Fladby, T.; Brønnick, K.; Aarsland, D.; Bonanni, L. Characterizing Resting-State EEG Oscillatory and Aperiodic Activity in Neurodegenerative Diseases: A Multicentric Study. *Computers in Biology and Medicine* **2025**, *197*, 111080. https://doi.org/10.1016/j.compbiomed.2025.111080.
25. Wen, H.; Liu, Z. Separating Fractal and Oscillatory Components in the Power Spectrum of Neurophysiological Signal. *Brain Topogr* **2016**, *29* (1), 13–26. https://doi.org/10.1007/s10548-015-0448-0.
